# An integrative toolbox to unlock the structure and dynamics of protein–surfactant complexes†

**DOI:** 10.1039/d0na00194e

**Published:** 2020-07-13

**Authors:** Adrian Sanchez-Fernandez, Carl Diehl, Judith E. Houston, Anna E. Leung, James P. Tellam, Sarah E. Rogers, Sylvain Prevost, Stefan Ulvenlund, Helen Sjögren, Marie Wahlgren

**Affiliations:** Food Technology, Engineering and Nutrition, Lund University Box 124 221 00 Lund Sweden adrian.sanchez-fernandez@food.lth.se; SARomics Biostructures AB Medicon Village, Scheelevägen 2 223 81 Lund Sweden; European Spallation Source Box 176 221 00 Lund Sweden; ISIS Neutron and Muon Source, Science and Technology Facilities Council, Rutherford Appleton Laboratory Didcot OX11 0QX UK; Institut Laue-Langevin 71 Avenue des Martyrs 38000 Grenoble France; EnzaBiotech AB Scheelevägen 22 223 63 Lund Sweden; Ferring Pharmaceuticals A/S Kay Fiskers Plads 11 2300 Copenhagen S Denmark

## Abstract

The interactions between protein and surfactants play an important role in the stability and performance of formulated products. Due to the high complexity of such interactions, multi-technique approaches are required to study these systems. Here, an integrative approach is used to investigate the various interactions in a model system composed of human growth hormone and sodium dodecyl sulfate. Contrast variation small-angle neutron scattering was used to obtain information on the structure of the protein, surfactant aggregates and surfactant–protein complexes. ^1^H and ^1^H–^13^C HSQC nuclear magnetic resonance spectroscopy was employed to probe the local structure and dynamics of specific amino acids upon surfactant addition. Through the combination of these advanced methods with fluorescence spectroscopy, circular dichroism and isothermal titration calorimetry, it was possible to identify the interaction mechanisms between the surfactant and the protein in the pre- and post-micellar regimes, and interconnect the results from different techniques. As such, the protein was revealed to evolve from a partially unfolded conformation at low SDS concentration to a molten globule at intermediate concentrations, where the protein conformation and local dynamics of hydrophobic amino acids are partially affected compared to the native state. At higher surfactant concentrations the local structure of the protein appears disrupted, and a decorated micelle structure is observed, where the protein is wrapped around a surfactant assembly. Importantly, this integrative approach allows for the identification of the characteristic fingerprints of complex transitions as seen by each technique, and establishes a methodology for an in-detail study of surfactant–protein systems.

## Abstract 2

A holistic approach is developed for investigating the interaction mechanism between proteins and surfactants in solution.

## Graphical abstract

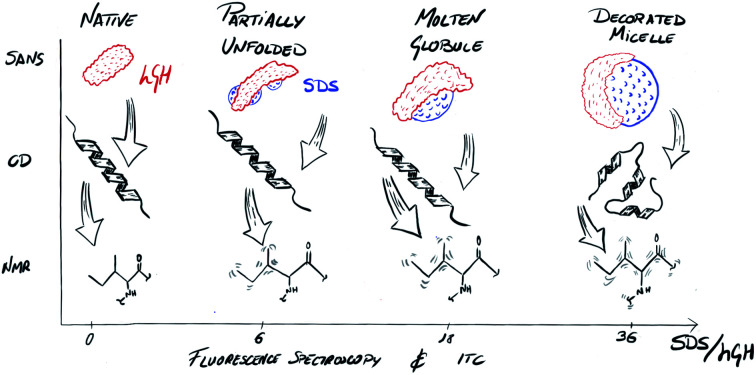

## Introduction

The interaction of proteins and surfactants in solution plays an essential role in a manifold of formulated products. It has been shown, for example, that the addition of surfactants is common practice in preparation of liquid pharmaceutical formulations, where the amphiphiles, generally polysorbates, are added in order to increase the stability and shelf-life of drug products.^1–3^ Due to this amphiphilic character, surfactant micelles and lipid bilayers are also able to stabilize otherwise insoluble proteins in aqueous solution by providing a membrane-like environment for the proteins.^4^ Furthermore, the combination of protein and surfactants in detergents provides a synergistic effect that enhances the detergency performance compared to that of surfactant-only systems.^5^ Other applications where surfactant–protein interactions are of great relevance include sodium dodecyl sulfate-polyacrylamide gel electrophoresis (SDS-PAGE), for the separation of biological macromolecules, drug delivery, and preservation of formulated food and cosmetic products.^6,7^ Due to the fundamental and applied importance of these interactions, researchers have been investigating surfactant–protein systems for almost a century,^8^ and still nowadays significant scientific activity is performed in the field.

Due to the complexity of the self-assembly behaviour and the formation of hybrid structures in surfactant–protein systems, the underlying phenomena of these interactions appear rather complicated and a general theory is still missing.^2,3,9^ It is widely accepted that ionic surfactants, such as sodium dodecyl sulfate (SDS), interact with proteins even at concentrations below the critical micelle concentration (CMC). These interactions arise from a synergistic contribution from the electrostatic attraction of the surfactant headgroup with oppositely charged protein residues combined with the hydrophobic forces around the protein hydrophobic domains.^10^ The adsorption of charged surfactant at low concentrations results in an alteration in the local charges of the protein, resulting in conformational and dynamic changes in the protein backbone. In contrast, non-ionic surfactants, *e.g.* dodecylmaltoside, show weaker interactions than ionic surfactants at equivalent surfactant-to-protein ratios, as in the absence of the electrostatic contribution the driving forces will be limited to hydrophobic and hydrogen bond interactions.^11^ Although binding at low surfactant concentration was also reported for non-ionics, these interactions are weak and were shown to not prompt significant changes in the protein structure. In this case, only minor changes in the protein structure are observed above the CMC, indicative of different mechanisms between charged and uncharged surfactants.^2,11,12^ The variety of interactions between proteins and surfactants often leads to diverse underlying changes – these may occur in parallel and sequential manners, alter the association–dissociation equilibria of the protein, involve electrostatic and/or hydrophobic forces, and result in the presence of coexistent structures in the colloidal domain (surfactant monomers, micelles, free proteins and surfactant–protein complexes).

The interaction between proteins and surfactants is therefore of inherent high complexity. As such, the study of these systems requires a systematic approach which often involves several techniques. Traditionally, analytical approaches combined spectroscopy, calorimetry and electrophoresis to study the binding between surfactant and protein, and the concomitant effects on the structure of proteins.^3,9^ Studies on kinetics of unfolding have been also shown to provide valuable information on the complexation mechanisms, especially above the surfactant CMC, where steady-state techniques provide limited information due to the micelle contribution to the signal.^3,13^ Scattering techniques, such as dynamic light scattering and small-angle scattering, have been used to prove the size and structure of protein surfactant complexes.^10,14,15^ Although the knowledge gathered through these techniques has greatly helped to understand interaction mechanisms from a structural perspective, the strong contribution from the surfactant monomers and micelles to the scattering often limits the information gained on the conformational state of the protein and complicates the elaboration of detailed structural models. Contrast variation in small-angle neutron scattering (SANS) has been used to determine structures of surfactant–protein complexes, showing that this approach can assess specific changes in protein conformation.^16–19^ Finally, computational simulations provide detailed information on the atomistic driving forces of these interactions, and complement the characterisation performed using experimental approaches.^20,21^

Somewhat surprisingly, advanced characterisation technologies (*e.g.* SANS) are rarely combined with lab-scale methods in properly integrated studies. Here a novel approach that combines advanced characterisation technologies – contrast variation SANS and protein nuclear magnetic resonance (NMR) spectroscopy – with routine methods – fluorescence spectroscopy (FS), circular dichroism spectroscopy (CD) and isothermal titration calorimetry (ITC) – to present a broad and interconnected characterisation of surfactant–protein interactions is presented. In particular, this methodology accesses information on the protein structure and local dynamics upon the addition of different amounts of surfactant. The system investigated comprised human growth hormone (hGH) and SDS. hGH is an important therapeutic protein yet prone to rapid denaturation and irreversible aggregation through interfacial stress.^22,23^ In combination with SDS, this protein shows various stages of interaction at different surfactant concentration. This opens the possibility to explore the various driving forces that promote the interaction between proteins and amphiphiles, as well as the impact on protein structure. Initially, the surfactant–protein system was characterised using in-house methodologies. The investigation continued with the advanced characterisation of the system using protein NMR and contrast variation SANS. Importantly, the outcome of this study establishes a new integrative approach to study these systems and links the results from in-house, lab-scale methods to those obtained from advanced characterisation techniques.

## Results and discussion

### Characterisation of structural transition points

**Fig. 1:**
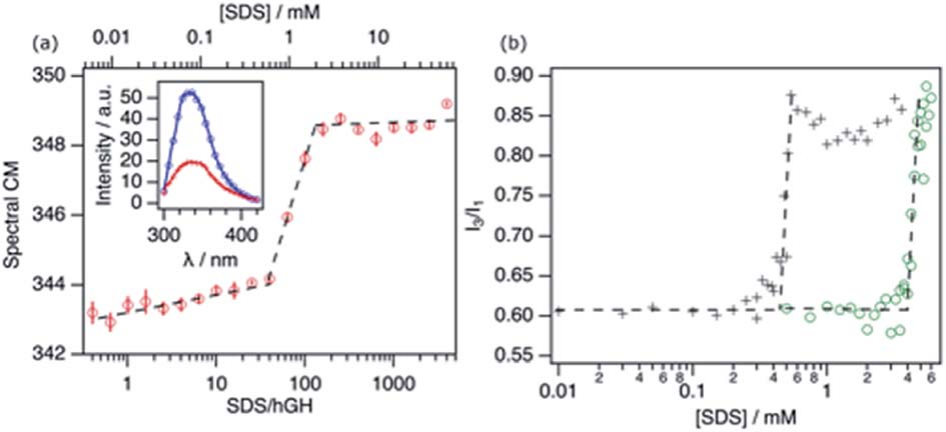
Characterisation of the structural transition points using fluorescence spectroscopy. (a) Spectral centre of mass of the Trp emission of 14.9 μM hGH at different protein/SDS ratios. The inset shows the spectrum of 16 (blue) and 344 (red) SDS molecules per hGH. (b) The ratio of *I*_3_/*I*_1_ from the emission spectra of pyrene with different concentrations of SDS in the absence (green circles) and presence (black crosses) of 10.3 μM hGH at different surfactant concentrations. The black dashed lines are added as guides, where the intersection corresponds to the aggregation concentration, CMC or CAC.

To determine the response of the protein to the addition of SDS, different techniques were combined. hGH is a small, globular protein, constituted by four antiparallel α-helices.^24^ The protein contains a single tryptophan (Trp) residue, which is embedded in the hydrophobic core of the protein in its native conformation. Therefore, the intrinsic fluorescence from this Trp residue is an adequate probe to track changes in the environment of the protein core. Fig. 1a shows the evolution of the spectral centre of mass (CM) of the fluorescence from hGH upon addition of SDS.

The emission spectrum of the pure protein has an emission maximum (*λ*_max_) at ∼338 nm, which correlates to the Trp residue being partially or totally embedded in a hydrophobic environment.^25^ Upon addition of surfactant, different stages can be identified corresponding to changes in the CM. Firstly, a gradual change in the CM upon addition of surfactant to *ca.* 38 surfactant-to-protein ratio is observed. Increasing the surfactant concentration beyond this value leads to a rapid increase in the CM, which correlates to the decreased emission intensity. Finally, beyond *ca.* 125 SDS/hGH ratio, the trend in the CM plateaus, where further addition of surfactant hardly changes the fluorescence spectrum. Although these changes are a clear indication of variations in the emission mode of the Trp residue, they are not as pronounced as for other systems where a strong shift to 350 nm is observed in the spectrum.^11,25^ The distinctive behaviour may be attributed to the fact that the Trp residue in hGH is already partially exposed to the solvent in the native state of the protein.^24,26^ As it has been previously shown, conformational changes in hGH induced by organic solvents (ethanol) or chemical denaturants (urea) that resulted in exposure to the solvent of the protein core, correlate with a decrease and red-shift of the Trp emission intensity.^27,28^ As such, these results can be interpreted as the unfolding of the protein and the exposure of hydrophobic residues to a more polar and heterogeneous environment.

The fluorescence response of pyrene (Pyr) at different concentrations of surfactant in the absence and presence of 10.3 μM of hGH is shown in Fig. 1b. The increase in the *I*_3_/*I*_1_ ratio, attributed to the formation of surfactant aggregates, is shifted to considerably lower concentrations in the presence of protein.^3,29^ For SDS in buffer solution in the absence of hGH, changes occur at 4.1 ± 0.3 mM, in agreement with previously published results of SDS micellisation in buffer.^10^ The addition of protein to the system shifts the inflection point to 0.43 ± 0.06 mM SDS, which corresponds to a surfactant-to-protein ratio of 42 ± 5. The considerably lower value of the critical association concentration (CAC) compared to the CMC is commonly attributed to the formation of micelle-like structures on the protein surface. This has been reported to occur at sub-micellar concentrations of SDS for various proteins.^10,11^

Various interaction stages between hGH and SDS were determined for different protein concentrations using ITC. As it has been previously shown,^18,25^ the concentration of surfactant required to reach a certain stage in the enthalpogram at various protein concentrations can be used to determine the concentration of unbound surfactant, [SDS]_unbound_, and the number of SDS molecules bound to the protein, *N*_agg_, as follows:[SDS] = [SDS]_unbound_ + *N*_agg_[hGH]

**Fig. 2:**
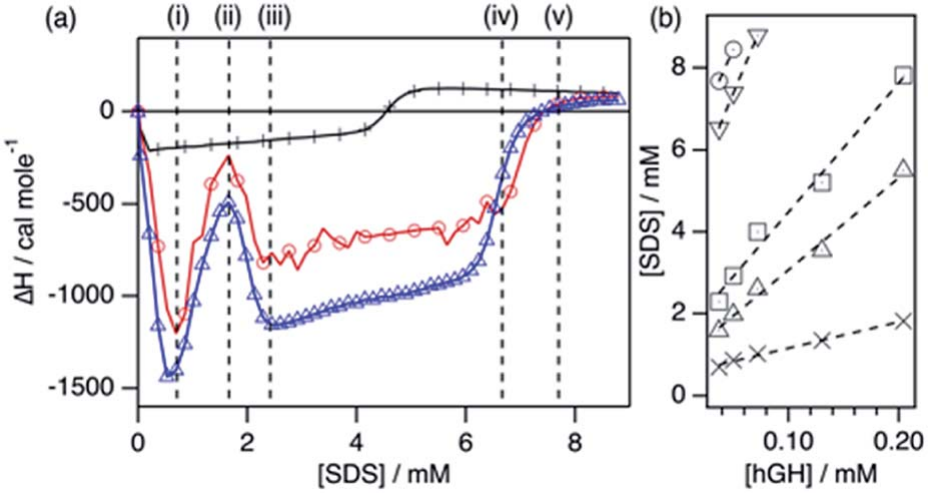
Results from the ITC characterisation. (a) Enthalpograms for the titration of hGH with SDS at 22 °C. Data is shown for the titration of 37 μM hGH with 60 mM SDS in buffered H_2_O (red circles) and 39 μM hGH with 60 mM d-SDS in buffered D_2_O (blue triangles). The data for the titration of SDS into the buffer (black crosses) is presented for comparison. (b) Fits of the transition points observed in the ITC results at different protein concentrations: (crosses) i, (upward triangles) ii, (squares) iii, (downward triangles) iv, and (circles) v.

The ITC enthalpograms for the surfactant–hGH system with the defined transitions are shown in Fig. 2 for one protein concentration in buffered H_2_O and D_2_O, together with the linear fits of the concentrations at which those transitions occur. The results from those fits at the different stages are presented in Table 1. The enthalpograms for the titration of other protein concentrations are included in the ESI.† Previous investigations of surfactant–protein interactions have shown that changes in the protein environment or structure have a characteristic fingerprint in an enthalpogram.^18,25^ A simplified rule of thumb is that the adsorption of surfactant molecules to the protein structure is an exothermic response, changes in protein conformation appear as endothermic signal and the formation of oligomers (dimer, trimer, …) is often exothermic. Nonetheless, the interpretation is not straightforward. In the raw enthalpogram, some injections initially present an endothermic response followed by a strong exothermic signal (see ESI†), and cannot easily be deconvoluted. As the integration of the heat was performed for each individual injection, and as such it contains both exo- and endothermic contributions to the signal, the separation of the underlying phenomena is not possible.

**Table 1:** Number of SDS molecules bound to hGH and concentration of unbound SDS at each stage as indicated in the enthalpogram. The total concentration of SDS for the 50 μM hGH system is included for reference

Transition	*N*_agg_	[SDS]_unbound_/mM	SDS/hGH_50μM_
i	6 ± 1	0.51 ± 0.04	17
ii	23 ± 1	0.81 ± 0.15	40
iii	32 ± 2	1.3 ± 0.3	58
iv	61 ± 2	4.1 ± 0.3	148
v	61 ± 5	5.4 ± 0.5	170

The enthalpograms show several interaction stages, which can be accounted for by the number of surfactant monomers associated with each protein molecule. As depicted in Table 1, each interaction stage relates to a different amount of bound surfactant molecules and the concentration of free surfactant, as calculated from the linear fits (Fig. 2b). (i) Upon the initial addition of surfactant, a significant shift to the exothermic region occurs, which is attributed to the adsorption of surfactant molecules onto the protein. This process appears to be initiated at the same surfactant concentration for all protein concentrations (see Fig. S3†). (ii) With further increasing the surfactant concentration, a decrease in the exothermic signal appears, which relates to the saturation of the process previously initiated. Also, these initial changes may reflect structural transitions on the protein induced by the adsorption of SDS, as the raw heat flow shows an endothermic contribution upon injection (see Fig. S2†). Nonetheless, the separation of transitions occurring in parallel cannot be performed by simply using calorimetry methods.^25^ (iii) This transition is followed by a strong, concentration-dependent shift to exothermic contributions. Once again, this probably reflects the adsorption of further surfactant molecules, which at this stage may be enabled by the previous change in protein conformation through the exposure of buried residues. (iv) The enthalpic signal then evolves to a switch in the trend, where the exothermic signal gradually decreases. This may relate to further protein structural transitions, but these must be probed using other techniques. Furthermore, this transition corresponds to an [SDS]_unbound_ of 4.1 mM and, thus, surfactant micelles are hypothesised to form at what it is referred to here as the effective surfactant CMC in the presence of protein (CMC_eff_). (v) Finally, the signal evolves to zero and no further changes are observed above this surfactant concentration. Interestingly, this transition corresponds to an [SDS]_unbound_ of 5.4 mM, which is higher than the CMC of the surfactant in the buffer. As SDS micellisation is a thermal at 22 °C, these final changes could be attributed to structural rearrangements or changes in the colloidal interactions, which occur in parallel to the formation of more free surfactant micelles. Above this transition point (v), no further changes happen to the protein.

One interesting observation can be made from the shape of the titration curves at different protein concentrations (Fig. S3†). In the protein concentration range studied here, the shape of the curves is similar, and the main difference appears in the surfactant concentration at which these transitions occur. This suggests that the transitions are mainly governed by specific protein–surfactant interactions at given surfactant-to-protein ratios, and not by non-specific interactions which occur at absolute surfactant concentrations.^25^

Protein NMR spectroscopy and SANS measurements need to be performed with deuterated compounds. Thus, an analogous ITC measurement was performed using d-SDS and buffered D_2_O to probe the effect of isotope substitution in the system. As observed in Fig. 2a, the enthalpogram of hGH in the presence of deuterated compounds shows a similar shape. Although the absolute values of enthalpy are shifted by ∼15%, the transition points appear at the same surfactant concentration. Thus, it can be concluded that the results from the measurements performed with isotopically substituted compounds appropriately describe the system with natural isotope abundance. This has been previously reported for other surfactant–protein systems.^18^

**Fig. 3:**
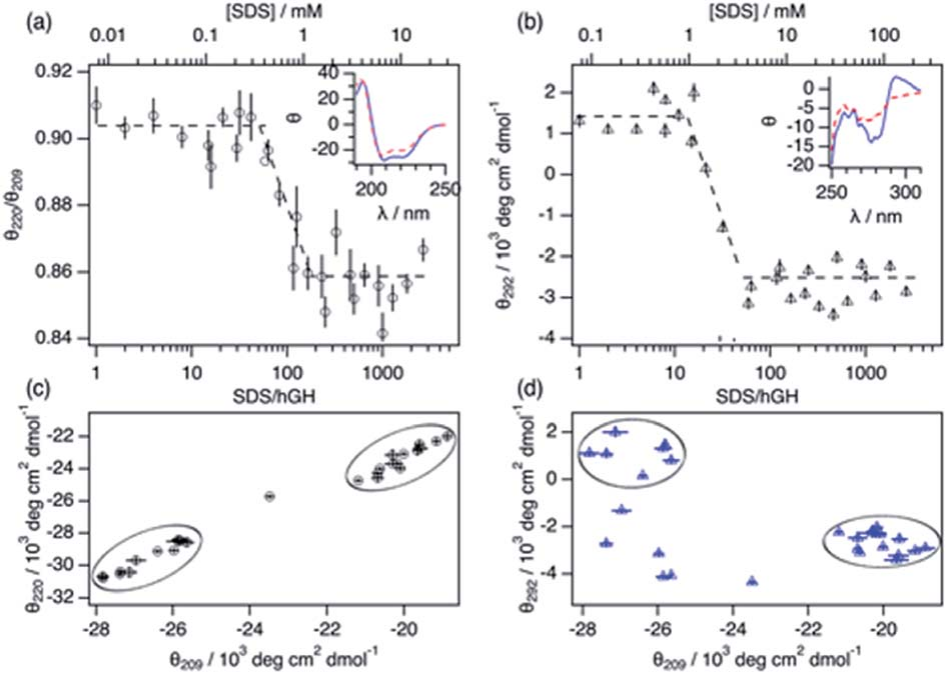
Characterisation of the structural transition points using circular dichroism. (a) Changes in the molar ellipticity ratio of hGH between 220 nm and 209 nm in the presence of SDS. The inset shows the characteristic far-UV CD spectra of 6.3 μM hGH with (blue solid lines) 15 and (red dashed lines) 408 SDS molecules per protein. (b) Changes in the 292 nm molar ellipticity of hGH at different concentrations of SDS. The inset shows the signal of 63 μM hGH with (blue solid line) 4 and (red dashed lines) 145 SDS molecules per protein. Parametric plots of the molar ellipticity: (c) 220 nm *vs.* 209 nm and (d) 292 nm *vs.* 209 nm. Black circles mark the initial and final populations.

Changes in protein secondary and tertiary structure upon addition of surfactant were investigated using CD. The results from the measurements in the far-UV and near-UV CD region are presented in Fig. 3. The secondary structure of the protein is mainly composed of a combination of α-helices and random coils, as reflected in the far-UV CD spectrum.^24^ The α-helix structure is characterised by two negative peaks at 209 and 222 nm and variations in the ratio between these two peaks (*θ*_220_/*θ*_209_) can be used to track changes in the secondary structure upon surfactant addition. Initially, the addition of surfactant does not appear to affect the secondary structure of the protein as *θ*_220_/*θ*_209_ remains constant within experimental error. At a surfactant-to-protein ratio of 59 ± 5, a decrease in the ellipticity ratio begins, which is attributed to changes in the secondary structure of the protein. This transition is completed around 162 ± 14 SDS/hGH, and further addition of surfactant above this concentration does not induce major changes in the signal. It should also be noted that the signal beyond this point still resembles that of a dominant α-helix structure. Thus, the secondary structure is only partially disrupted at this stage and no changes occur at higher surfactant concentrations. The tertiary structure of the protein goes through a similar transition but at significantly lower surfactant-to-protein ratio. The signal at 292 nm, attributed to the chiral influence of the Trp environment, drops at a surfactant/protein ratio of 16 ± 2 and levels off at around 58 ± 4. The remaining spectral features around 265 nm suggest some tertiary structure is preserved, potentially due to the resilience of the disulphide bonds. These data confirm that the disruption of the structure occurs following a two-stage mechanism. As shown in the parametric plots (Fig. 3c and d), the secondary and tertiary structure remain similar to those of the native structure at low surfactant content, which then evolves to an intermediate state where the tertiary structure is partially disrupted. Further increase in surfactant concentration results in changes in the secondary structure whilst the tertiary structure does not go through further changes.

The combination of spectroscopy and calorimetry techniques supports the identification of characteristic transitions occurring in the system upon surfactant addition. The limited availability of advanced characterisation technologies significantly reduces the number of samples that can be measured. Therefore, in-house characterisation using ITC, fluorescence and CD has been used to identify regions of interest for the investigation and to select the samples to be characterised by SANS and NMR spectroscopy. These samples were selected as follows: protein native state, early adsorption of SDS, disruption of tertiary structure, formation of SDS clusters and disruption of secondary structure, and above CMC_eff_.

### Local dynamics and structure

**Fig. 4:**
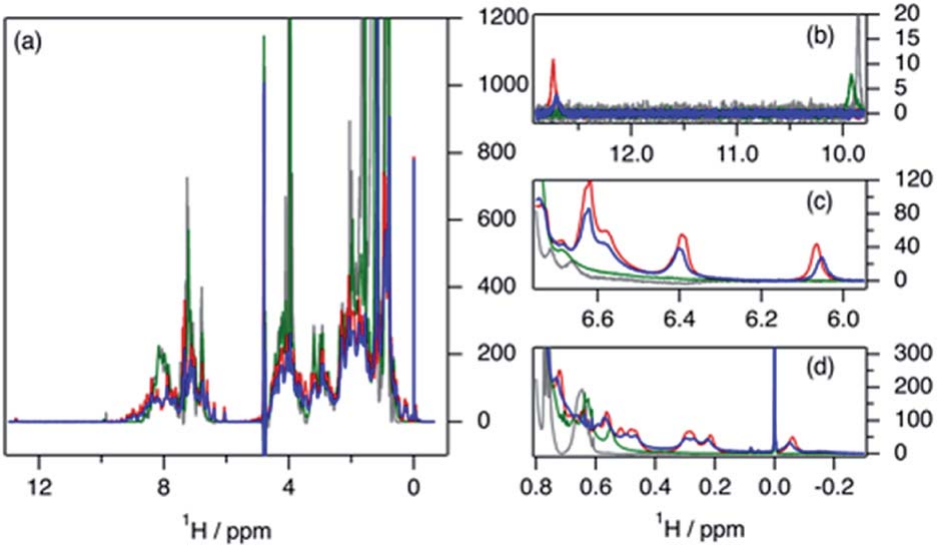
^1^H NMR spectra of 0.389 mM hGH in the presence of SDS at different surfactant–protein ratios: (blue) 0, (red) 4, (green) 37 and (grey) 360. The full spectra are shown in (a). The subplots represent the expanded regions of (b) tryptophan, (c) tyrosine, and (d) methyl.

The variation in local structure and dynamics of hGH in the presence of SDS has been studied using NMR spectroscopy. ^1^H NMR spectra of the protein at different surfactant concentrations are presented in Fig. 4. Assignments for some aromatic and aliphatic sidechains, as well as the backbone amide resonances at two different pH, have been previously published.^30,31^ However, to the best of our knowledge, no full assignment of the methyl region has been performed and, therefore, atom-specific assignments could not be performed on the acquired spectra. Due to such a limitation, the analysis of the environment of specific residues could not be performed. However, based on reported assignments, some tentative assignments are performed for aromatic sidechain peaks in the ^1^H NMR spectra of hGH in the absence of SDS. The peak at 12.75 ppm in the ^1^H NMR spectrum is consistent with the presence of a unique tryptophan residue in hGH. Based on the crystal structure, it is known that the H-epsilon of Trp86 forms a well-defined hydrogen bond with Asp169 causing significant upshift of peak position.^24^ Similarly, based on reported assignments, some amino acid sidechains can be identified in the ^1^H spectra in addition to Trp86, namely two Tyr and four Leu, Val and Ile sidechains.^30,31^

The evaluation of ^1^H NMR spectra at different SDS concentrations gives information on the higher-order structure (HOS) of the protein, as well as changes in the amino acid residue environment. Overall, there is very little change in HOS for hGH in native and SDS/hGH = 4, although the peak intensity is generally lower in the presence of surfactant, indicating a higher transverse relaxation rate due to conformational exchange. The assigned sidechains all show small but significant changes in peak position and intensity upon the initial addition of SDS.

In particular, the Trp86 sidechain gets significantly broadened and downshifted, indicating a subtle change in this residue's environment. Among the sidechains identified, only Trp86 can be unambiguously located to the hydrophobic core of hGH, based on surface accessible area.^24^ Therefore, it can be concluded that the adsorption of the surfactant at this surfactant–protein ratio takes place at the protein core, whilst the overall environment of the sidechain residues does not suffer major changes when compared to the native state. A more drastic shift in ^1^H spectra is seen for SDS/hGH = 37. Overall, the ^1^H NMR spectra show a narrower chemical shift dispersion with sharper peaks, indicating that hGH is significantly unfolded and local dynamics are increased. When tracking changes in the Trp86 peak, a peak at 9.92 ppm appears, which corresponds to the exposure of H-epsilon residues to a polar environment based on reference chemical shifts.^30^ At SDS/hGH = 360, the spectrum shows further changes. There are some peaks with narrow peak widths indicating further unfolding of hGH. In particular, this can be readily seen for the sidechain of Trp86, which has a sharper peak and is slightly downshifted compared to the spectrum for SDS/hGH = 37. It can also be seen that the signal from the surfactant starts to dominate the relaxation in the methyl region and, as consequence, changes in this region cannot be interpreted from this spectrum.

**Fig. 5:**
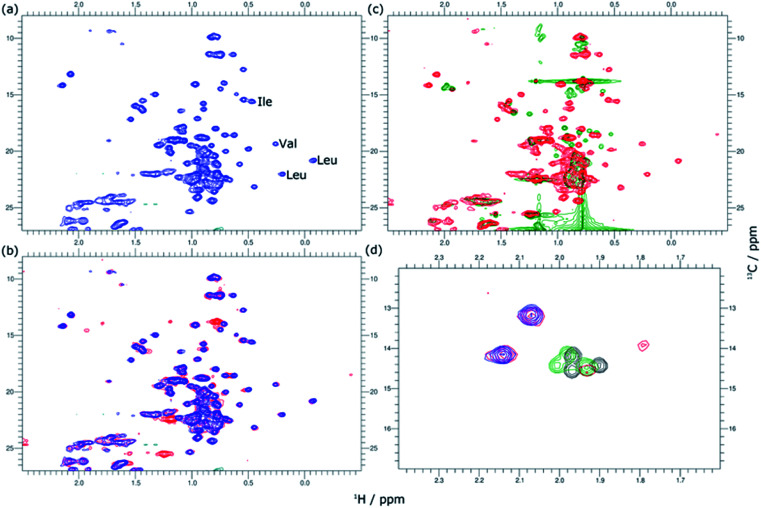
2D ^1^H–^13^C HSQC spectra of 0.389 mM hGH in the presence of SDS at different surfactant–protein ratios. (a) Full spectrum of the native protein, (blue) SDS/hGH = 0, (b) superimposed (blue) native protein signal and signal from (red) SDS/hGH = 4, and (c) superimposed (red) SDS/hGH = 37 signal and signal from (green) SDS/hGH = 37. (d) Overlaid signals in the methionine region of SDS/hGH at SDS/hGH ratios of (blue) 0, (re) 4, (green) 37, and (grey) 360.

2D ^1^H–^13^C HSQC spectra for hGH at different SDS concentrations confirm this picture. The data from these measurements are presented in Fig. 5. Based on known assignments, some residues can be identified as Leu, Val or Ile sidechain. For 2D methyl spectrum, the terminal methyl groups of methionines are typically well resolved. There are three methionines in hGH, all located in the hydrophobic core as based on surface accessible area,^24^ two of which can be easily identified in the ^1^H–^13^C HSQC spectra. Thus, the ^1^H–^13^C HSQC spectrum shows well-resolved peaks for the protein core and these serve as a fingerprint for the protein conformational state.

In Fig. 5, methyl peaks belonging to SDS can be readily identified in the spectrum, *ca.* 0.8 ppm, with no major overlap with protein methyl peaks. In the protein signal there are only small differences in peaks between the native state and upon addition of 4 SDS molecules per hGH, with some peaks showing a slight shift in the presence of surfactant. Overall, the ^13^C-HSQC spectrum is very similar, showing a comparable HOS between the native structure and the low SDS concentration complex. Interestingly, some peaks appear in the methionine region, indicating the presence of a slow environment exchange for methionines. As observed for ^1^H NMR spectra, there is a more drastic change at 37 SDS/hGH, where a change to sharper shift dispersion of the peaks can be seen for the methyl groups. At this surfactant concentration, there is a significant effect on the spectrum from SDS due to the intense contribution from the surfactant. Based on the ^13^C-HSQC, hGH is likely highly unfolded, although some residual structure remains based on peak overlaps between 4 and 37 SDS/hGH ratios. A more detailed evaluation of the methionine region clearly shows resolved peaks for the three methionines of hGH, which are shifted from the native structure and show that the HOS for SDS/hGH = 37 is significantly altered. At the highest surfactant content, SDS/hGH = 360, only a few peaks can be resolved in the ^13^C-HSQC spectrum due to the strong SDS signal. From the meagre spectral information, the methionine region can still be resolved, and it is seen that the three methionine peaks are slightly shifted compared to SDS/hGH = 37. Thus, further structural rearrangement appears at this surfactant concentration.

The peak changes detected above can also be correlated to the crystal structure of hGH, where Met14, Trp86, Met125 and Met170 are all located in different α-helices of the protein, which are all contained within the hydrophobic core in the protein native state.^24^ Minor peak changes are detected for these sidechains at low SDS/hGH ratio, indicating that the adsorption of surfactant onto the protein core results in subtle environment changes whilst the HOS remains relatively unchanged. When the concentration of SDS is further increased, a significant amount of order and rigidity in the protein is lost, thereby indicating that SDS shifts the equilibrium towards an unfolded state.

### Conformational landscape

**Fig. 6:**
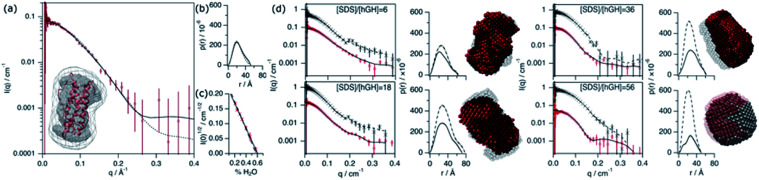
SANS characterisation of the conformational state of the protein and surfactant–protein complex. (a) SANS curve and best fits (solid line – IFT; dashed line – CRYSON) of 0.154 mM hGH in D_2_O, 10 mM phosphate buffer. The inset shows the superposition of the protein crystal structure and the scattering volume calculated using DAMMIN. The scattering volume was averaged from 8 generated structures, where the grey dummy atom model shows the most-likely populated volume between the different models and the grey-map contour represents the spread from this averaged volume. (b) Pair distance-distribution function of the protein calculated using IFT (solid line) and using crystal structure (dashed lines). (c) Experimental (solid line) and calculated (dashed line) square root of the forward scattering intensity *versus* fraction of H_2_O in the solvent used to calculate the contrast match point. (d) SANS data and best IFT fits, *p*(*r*) and bead models at different surfactant–protein concentrations, as indicated in each graph. Results are presented for protein and complex contrast and depicted as follows: protein contrast: SANS data – red circles, IFT fits and *p*(*r*) – solid lines, and bead model – red spheres. Complex contrast: SANS data – black crosses, IFT fits and *p*(*r*) – dashed lines, and bead model – grey spheres. The transparency of the models was tuned to ease the interpretation. Data collected on SANS2d.

The conformation of the native protein in D_2_O buffer was determined using SANS. In brief, the pair distance-distribution function of the scatterer (*p*(*r*)) was initially determined through the IFT approach.^32^ The resulting *p*(*r*) of the protein, together with the scattering data, were used to calculate *ab initio* bead models using DAMMIN, which was subsequently averaged using SUPCOMB.^33,34^ Finally, the scattering of the crystal structure was calculated and fitted using the CRYSON software.^35^ The scattering length density (SLD) of the protein was determined through a contrast matching experiment. For this experiment, 0.150 mM hGH was measured at different H_2_O/D_2_O ratios and the forward scattering (*I*(0)) was determined through the Guinier approximation.^36^ The contrast match point of the protein can be determined from the *x*-intercept of a linear fit of the square-root of *I*(0) *versus* the H_2_O/D_2_O ratio. From the value of the intercept with the *x*-axis, the SLD of the protein under these conditions is calculated. Experimental data, fits, the *ab initio* model, and contrast variation results are presented in Fig. 6.

This SANS characterisation has shown that the protein adopts a globular morphology in solution and the experimental scattering of the protein is in good agreement with the predicted scattering from the crystal structure. The *D*_max_ and *R*_g_ of the scatterer were found to be 51.7 Å and 18.4 ± 0.3 Å, respectively, and the same dimensions predicted from the crystal structure are 52 Å and 17.4 Å. The contrast match point of the protein is 60.1 ± 2.1% H_2_O (SLD = 2.31 × 10^−6^ ± 0.08 × 10^−6^ Å^−2^), similar to the theoretical value, 57.25% H_2_O (SLD = 2.41 × 10^−6^ Å^−2^), calculated using the amino acid sequence and estimating a 90% exchange of exchangeable hydrogens.^37^ The subtle difference may arise from a difference in the number of exposed hydrogens that are exchangeable with the solvent.

Four different isotopic mixtures were used to follow the structural changes occurring in the system and to elaborate a detailed model of interaction between the protein and surfactant using SANS. Each of these contrasts uses a specific isotopic labelling scheme to focus on a particular feature of the systems: surfactant–solvent contrast matched – protein visible (protein contrast) , surfactant–protein contrast matched – complex visible (complex contrast), protein–solvent contrast matched – surfactant visible (surfactant contrast), and zero-average contrast (ZAC) condition (ZAC contrast). Table 2 shows the SLD of each component of the system used for the contrastvariation experiment. Protein contrast discriminates the scattering from the surfactant, focusing on the signal from the protein, which can thus be used to follow the conformational state of this. Complex contrast shows the structural features of the complex formed between surfactant molecules and protein. The existence of internal scattering density correlations has been previously shown to complicate the analysis of data through the IFT method.^38^ However, using the contrast matching approach, where the SLD of the surfactant equals that of the protein, internal scattering length density correlations vanish and, thus, this contrast can be used to unambiguously depict the structure of the complex. Surfactant contrasts focuses on the scattering from the surfactant and thus provides information on the structure of SDS assembles in the mesoscopic scale. Finally, the ZAC contrast uses an specific contrast condition from which two theoretical predictions can be done: (1) the formation of a complex will lead to no effective forward scattering because of the contrast match condition (*I*(0) = 0), (2) if segregation of compounds occur within the complex (*e.g.* formation of a surfactant cluster), a broad peak will appear from the density correlation between the complex domains.^39,40^ Further information on the contrast conditions used here can be found in the ESI.† The SANS data, IFT fits, *p*(*r*) and *ab initio* models for these two contrasts are presented in Fig. 6.

**Table 2:** SLDs for each of the components used in the contrast variation SANS experiments

Contrast	Protein	Complex	Surfactant	ZAC
SLD_hGH_/×10^−6^ Å^−2^	3.09	3.09	2.31	ZAC^a^
SLD_SDS_/×10^−6^ Å^−2^	6.37	3.09	6.37	6.45
SLD_Solv_/×10^−6^ Å^−2^	6.37	6.37	2.31	ZAC^b^

a The SLD of the protein depends on the SLD of the solvent and was calculated for each sample from the contrast-match experiment results.

b The SLD of the solvent is calculated using the equation for the ZAC condition (see ESI).

**Fig. 7:**
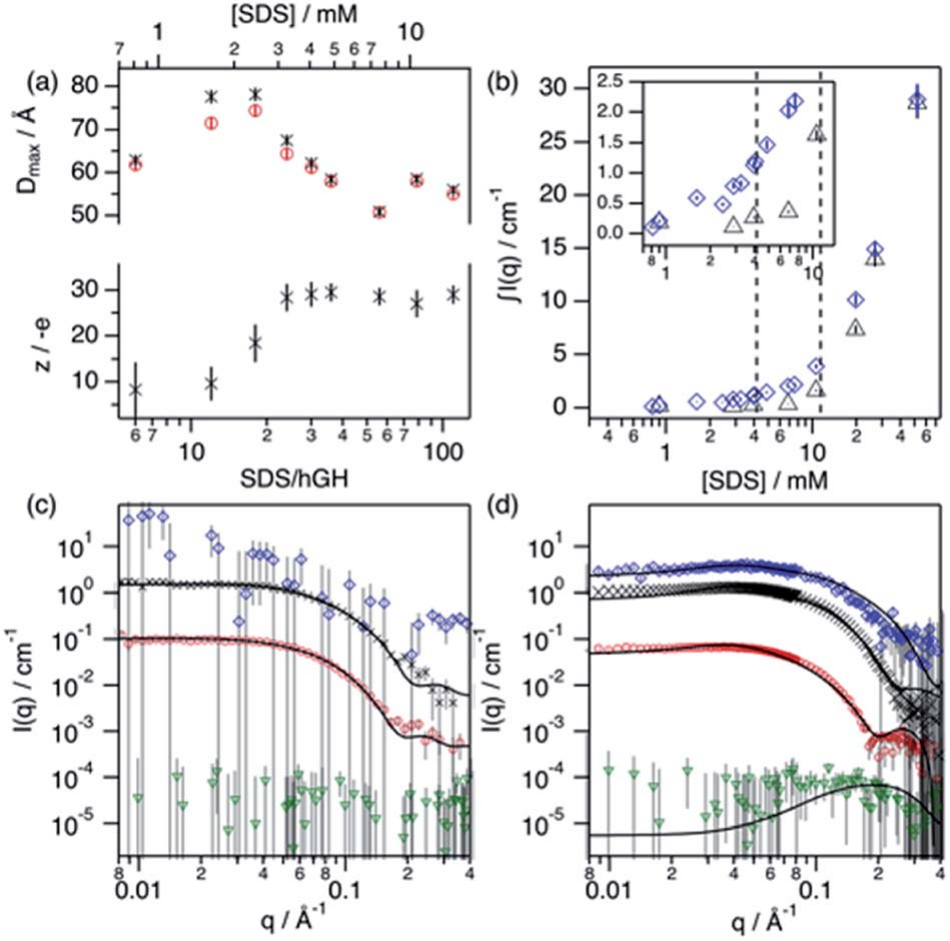
Results from the analysis of SANS data. (a) *D*_max_ and particle charge values for the protein (red circles) and complex (black crosses) upon addition of SDS. (b) Integrated scattered intensity at different surfactant concentrations in the absence (black triangles) and presence (blue diamonds) of 0.135 mM hGH. The inset shows the expanded region where the CAC and CMC_eff_ are located, as indicated by the dashed lines. SANS data and best fits of (c) SDS/hGH = 18 and (d) SDS/hGH = 56 at 0.135 mM hGH in different contrasts: protein contrast (red circles), complex contrast (black crosses), surfactant contrast (blue diamonds) and ZAC contrast (green triangles). Solid lines show best fits to the data. Data and fits were offset for clarity. Data collected on SANS2d (c) and D11 (d).

Upon addition of surfactant, the maximum dimension of the scatterer differs from that of the native protein structure (*D*_max_ = 51.7 Å) and free surfactant micelles (*D*_max_ = 48.4 Å), indicating that a complexed structure is formed. The evolution of the main structural parameters with surfactant content is shown in Fig. 7a. The calculated *p*(*r*) for protein and complex initially shows similar globular morphologies and sizes at SDS/hGH = 6, indicating that at low SDS concentration surfactant adsorption occurs and that the conformation of the protein upon complexation is slightly changed from that in its native state, potentially with some surfactant monomers sparsely adsorbed. When the surfactant ratio is increased to 12 and 18, the size of the complex gradually increases but remains globular. The largest structure was identified at SDS/hGH = 18, where the protein adopts a more unfolded state. Here, the surfactant still appears to be adsorbed onto the protein in a disorderly fashion. Further increasing the ratio to SDS/hGH = 36 results in shrinkage, where both the protein and the complex show the same *D*_max_. At this concentration, the dummy bead model of the protein appears to partially occupy the complex volume, leaving large clumps of the complex occupied by surfactant clusters. A significant change in the morphology of the protein is observed at SDS/hGH = 56. From the *p*(*r*) of the complex it appears to retain its spheroidal morphology, while the protein *p*(*r*) now shows a shoulder around 12 Å in the real space function. This is attributed to the appearance of a correlation length in the scatterer of such a magnitude, which is not present at lower surfactant concentrations. From the corresponding dummy bead model, the complex effectively adopts a spheroidal morphology, where the protein sits at the surface forming a shell. Thus, the characteristic dimensions of the protein can be described as the outer diameter of the spherical shell, which in turn is the *D*_max_ of the scatterer, and the thickness of such a shell, which relates to the shoulder in the protein *p*(*r*). At the highest SDS concentration measured below the CMC_eff_, which corresponds to an SDS/hGH ratio of 149, the complex mostly retains the core–shell structure previously observed. However, the increase in surfactant concentration results in a change in size of the complex, as observed in Fig. 7, which may suggest that the adsorption of surfactant takes place at the core of the complex.

At concentrations above the CMC_eff_ the data from complex contrast is affected by the contribution of free SDS micelles in solution and, thus, no structural characteristics of the complex can be probed. However, the signal from protein contrast can still be used for extracting information about the protein conformation at high SDS concentrations. The results from our measurements above the CMC_eff_ show that the protein retains its partially unfolded, thin-shell morphology up to relatively high surfactant concentrations (27 mM).

Surfactant contrast, where the solvent and the protein are contrast matched, provides details of the surfactant arrangement when complexed with the protein. Fig. 7b shows the integrated scattered intensity (∫*I*(*q*)) for surfactant contrast at different surfactant concentrations in the absence and presence of protein. The scattering curves of this contrast are presented in the ESI.† Different regions in the plot can be identified as follows: initially, the signal arising from SDS is barely above the noise of the measurement (∼0.3 cm^−1^) and no information other than the absence of significant surfactant aggregation can be extracted. This suggests that in this concentration range the system is below the CAC.

With increasing surfactant concentration, the signal for surfactant contrast gradually increases, but it remains too low to determine structural features from these. However, some meagre details can be observed: the integrated scattering intensity increases with the addition of more surfactant, such an increase is more pronounced in the presence of protein than in the absence of protein, and the scattered signal comes from seemingly small spheroidal aggregates. This suggests that the aggregation of surfactant is favoured in the presence of protein, the system is above the CAC and the surfactant aggregates are loosely ordered. At concentrations around the CMC_eff_, the surfactant scattering significantly increases resembling that of spheroids interacting electrostatically. Due to the expected low concentration of free surfactant (surfactant monomers in solution or free surfactant micelles), as shown by ITC, this signal must originate from surfactant clusters complexed with the protein. When the SDS concentration is much higher than the CMC, no clear structural transitions are observed in the surfactant phase and the scattering is probably dominated by the presence of surfactant micelles.

Following changes in the trends of ∫*I*(*q*), surfactant contrast can be used to track the formation of surfactant aggregates in the absence and presence of proteins. Despite of the lack of resolution due to the limited number of samples measured using SANS, and the poor signal-to-noise ratio at low surfactant concentrations, approximate values can be obtained and these are in good agreement with those obtained through the ITC experiments (ITC − CAC = 3.9 mM, CMC = 13.6 mM; SANS − CAC = 4 ± 1 mM, CMC = 11 ± 2 mM for 0.135 mM hGH).

In order to validate the models, a simultaneous fit of the four neutron contrasts was performed for the complex at low (18) and high (56) SDS/hGH ratio. The data and best fits are presented in Fig. 7. Visual inspection of the data reveals that the shape of the scattering curves significantly differs between the two systems, confirming that these represent different stages of interaction between the protein and the surfactant. The most relevant results are summarised here, with a more detailed analysis presented in the ESI.†

The low surfactant concentration was satisfactorily fit using a uniform ellipsoid form factor and the RMSA (Fig. 7c).^41,42^ At 18 surfactant/protein ratio, complex contrast shows a slightly larger size than the protein upon complexation with the surfactant, protein contrast, and the latter appears slightly larger than the native structure. The signal from surfactant contrast is very weak, indicating the absence of significant surfactant aggregation and the adsorption of sparse surfactant molecules onto the protein. For this surfactant concentration, ZAC contrast shows effectively no signal, which in turn relates to the formation of a surfactant–protein complex that cancels out the forward scattering. Also, the absence of a correlation peak in the ZAC contrast indicates the absence of a well-defined segregated domain within the complex. From the structure factor calculations, the charge of the complex is shown to evolve upon surfactant complexation. At this SDS/hGH ratio, the complex charge evolves to −15 ± 6 net charge from the native charge of the protein at pH 7, −5 ± 1. This charge evolution is attributed to the complexation of protein with negatively charged surfactant monomers and agrees (within the error) with the *N*_agg_ obtained through ITC.

The SANS data of hGH with a high concentration of SDS was best described using a core–shell ellipsoid, as previously shown by the IFT analysis. When simultaneously fitting the four neutron contrasts, it is observed that the protein (protein contrast) is situated at the shell of the spheroidal complex (complex contrast), whereas the surfactant molecules gather at the centre of the complex forming a micelle-like structure (surfactant contrast). From the resulting core volume, the number of SDS molecules associated to the complex was calculated as *N*_agg_ = *v*_core_/*v*_SDS_. This surfactant assembly was found to be smaller than free surfactant micelles and the *N*_agg_ is lower than that of SDS micelles (ITC − *N*_agg_ = 32, SANS − *N*_agg_ = 26). The ZAC shows effectively no forward scattering, as expected from the formation of a complex (ZAC contrast). However, a bump is observed at high *q* (∼0.2 Å^−1^), unlike in the low concentration sample. From the theoretical prediction of the ZAC condition, this bump correlates with the existence of a short-range density correlation, which arises from the SLD difference between the surfactant micelle (SLD_SDS_ = 6.45 × 10^−6^ Å^−2^) and the protein (SLD_hGH,0.75D_2_O_ = 2.79 × 10^−6^ Å^−2^). When co-refining the neutron contrasts to the core–shell structure it was required to fit the SLD of the shell instead of fixing it to the SLD of the protein in order to satisfy all contrasts. This can be interpreted as a partial coverage of the micelle by the protein, instead of full coverage. Using such an approach, the shell SLD was 3.43 × 10^−6^ Å^−2^, resulting in a coverage of *ca*. 66%, and providing a robust fit for all of the contrasts. This is in good agreement with computational studies that show which show a non-uniform coverage of SDS micelles by the protein, wrapping the micelle at the head–tail interface.^43^ In terms of interparticle interaction, a subtle peak at ∼0.04 Å^−1^ results from the stronger electrostatic repulsion between particles in solution compared to that at lower SDS concentration. The net charge of the complex was 29 ± 4 negative charges, compared to −15 ± 6 observed a the lower SDS concentration, confirming the increase in particle charge due to the complexation of SDS molecules with the protein. This, again, is relatively close to the previously determined *N*_agg_ and the differences may rely on charge neutralisation upon interaction with positively charged residues and/or counterion condensation.

### An integrative approach to reveal the interaction mechanism

**Fig. 8:**
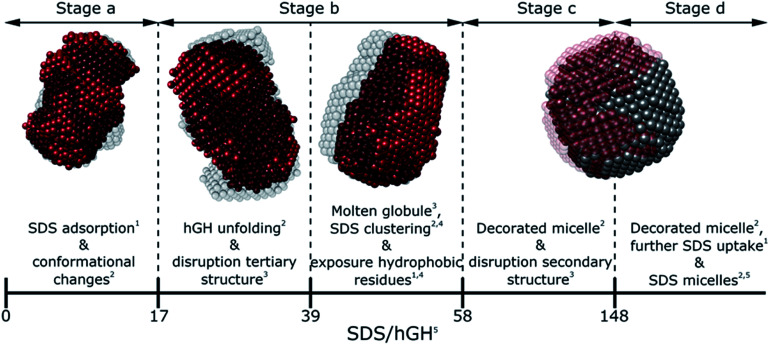
Schematic model summarising the different interaction stages between SDS and hGH as mainly seen by each technique: NMR,^1^ contrast variation SANS,^2^ CD,^3^ fluorescence^4^ spectroscopy and ITC.^5^

Generally, SDS shows a strong interacting character with soluble proteins even at low surfactant concentration, capable of modifying secondary and tertiary structure, conformation, self-association and stability of the biomolecule.^3,10,18^ Furthermore, different interaction mechanisms with varied concentration of SDS relate to a wide range of surfactant–protein complexes and intermediates.^44^ A general model for these mechanisms between surfactants and proteins can be developed through the combination of the techniques presented here, where the characteristic transitions can be used to identify specific changes in protein structure and dynamics. The surfactant–protein ratios at which the transitions are observed for the hGH-SDS system by each technique are summarised in Fig. 8 and Table 3.

**Table 3:** Surfactant–protein ratios at which different transitions (i–v) between different stages occur, as seen by each technique

	i	ii	iii	iv	v
Technique	SDS/hGH				
ITC	17 ± 2	39 ± 3	58 ± 5	148 ± 5	173 ± 3
Trp fluorescence		38 ± 3		125 ± 15	
Pyr fluorescence		42 ± 4			
Near CD	16 ± 4		59 ± 4		
Far CD			52 ± 6	142 ± 15	
SANS	18	36	56	149	

#### Stage a (below transition i) – SDS/hGH < 17

At low surfactant concentration, calorimetry measurements show that early SDS binding to the protein occurs, with aggregation numbers ranging from 1 to 6 SDS molecules per protein. As observed in the near-UV CD results, no major changes in the environment of amino acid residues are observed and the tertiary structure of the protein remains largely unaffected. However, SANS results show subtle variations in protein conformation, as seen by changes in the *R*_g_ of the protein, and the formation of a surfactant–protein complex with sparsely adsorbed surfactant monomers. These surfactants are also shown to affect the local environment of specific amino acids; in particular, NMR spectroscopy shows small changes in chemical shifts of those amino acids located at the protein core. Therefore, hydrophobic interactions between the protein hydrophobic pockets and the surfactant tail play a significant role in the adsorption at low surfactant concentration, whilst the environment of tryptophan is mostly retained and still surrounded by non-polar molecules, *e.g.* hydrophobic amino acids and surfactant tails. The early interaction of SDS monomers with proteins can also be attributed to electrostatic binding with positively charged residues, which synergistically contributes to the hydrophobic interactions and promotes early surfactant–protein complexation.^3,10,18^ In contrast, non-ionic surfactants, where the interaction is mainly driven by a balance between hydrophobic interactions (attractive) and steric hindrance (repulsive), has been repeatedly shown to weakly interact at sub-CMC concentrations.^3,11^

#### Stage b (between transitions i and ii) – 17 < SDS/hGH < 58

Further addition of SDS initiates a change in the tertiary structure of the protein at *ca.* 16 SDS/hGH ratio, whilst the secondary structure of the protein remains unaffected. Such a transition is clearly observed in the near-UV region of the CD spectrum and correlates to the change in the trends observed in the enthalpogram obtained by ITC. At this stage, a significant variation in protein conformation is observed by SANS, which appears as a second-order transition as observed in the parametric plot of the near-UV CD data. This unfolding results in a significant change in the local environment and dynamics of the protein core, where the Trp residue is exposed to a more polar environment at *ca.* 38 SDS/hGH, as judged by the fluorescence and 1D ^1^H-NMR spectroscopy measurements. Also, 1D and 2D NMR spectra show variations in the chemical shifts and peak intensity of hydrophobic sidechains due to changes in protein conformation and dynamics. This intermediate complex also shows well defined hydrophobic pockets, as shown by pyrene assay and SANS results, where the surfactant forms clusters on the protein surface, with an aggregation number of 23 SDS molecules. At this stage, the formation of a charge-stabilised molten globule is hypothesised. The formation of such an intermediate has been previously reported for hGH in the presence of a chemical denaturant and non-ionic surfactants,^45^ where the native tertiary structure of the protein is disrupted whilst the secondary structure is retained.

#### Stage c (between transitions ii and iii) – 58 < SDS/hGH < 148

When the SDS/hGH ratio is increased to *ca.* 58, the changes in the tertiary structure get to completion, although it is not totally disrupted as shown by the near-UV CD results. The unfolding of the protein results in the exposure of new binding sites, as the uptake of surfactant increases to 32 SDS molecules per protein, which correlates to the second exothermic transition in the enthalpogram. This transition from the previous unstructured surfactant clusters to micelle-like structures is potentially driven by the conformational entropy of the surfactant, where the formation of defined hydrophobic domains becomes more favourable above a certain aggregation number.^46^ At this stage, the secondary structure of the protein begins to decrease in α-helix content, as a subtle increase in the random coil content is observed. This two-stage denaturation mechanism has been previously shown for proteins containing α-helices.^10,11,44^ Contrast variation SANS revealed an interesting structural transition in the system at this stage. Whilst the complex retains the globular structure, the protein conformation changes from an unfolded, ellipsoidal conformation to a thin shell at the interface between the complex and the solvent. This structure has been previously reported for several proteins in the presence of SDS as a “decorated micelle”.^10,17,47^ The addition of more SDS up to a ratio of *ca.* 148 does not result in further significant structural changes. From ITC results it is seen that more surfactant molecules attach to the complex, resulting in the small increase in size observed through SANS. However, the overall structure of the cluster is preserved, and the protein remains adsorbed at the cluster surface.

#### Stage d (above transition iv) – SDS/hGH > 148

At higher surfactant concentration, *ca*. >12 mM, the formation of free surfactant micelles is observed. Benchtop steady-state techniques have been found to provide limited information on protein transitions above the CMC_eff_, as protein interacting sites seem to be saturated and protein secondary and tertiary structure do not present major rearrangements. Furthermore, small-angle X-ray scattering and light scattering are affected by the strong contribution of free micelles to the signal.^3^ Therefore, studying protein changes in this regime has often been challenging with these scattering techniques. However, contrast variation SANS and NMR spectroscopy provide information on the protein characteristics even at high surfactant concentration. From the results collected here, it is seen that the protein structure does not show significant structural variations up to SDS/hGH = 296, the maximum surfactant-to-protein ratio tested, and the decorated micelle conformation is still observed. Small variations are however visible in the NMR spectra, which could relate to subtle changes in local structure and/or dynamics of the hGH amino acids. It is, however, important to note that micelle morphology may affect the structure of the protein, but as this investigation has been focused on concentrations that range from monomeric SDS to spheroidal surfactant micelles, this effect has not been studied (see ESI†). At higher SDS concentrations, where micelle sphere-to-rod transition occurs, micelle curvature may cause further conformational changes in the protein.^48^

The various interaction mechanisms between SDS and hGH described here are driven by numerous forces. Although surfactant–protein and surfactant–surfactant hydrophobic interactions appear to play a role in the whole concentration range studied, other forces may be more specific at certain surfactant–protein ratios. At low SDS concentrations, hydrophobic interactions between the core of the protein and surfactant tail, together with electrostatic binding strongly influences the adsorption of SDS to hGH and results in early complexation. When the surfactant concentration increases, the formation of transient SDS clusters is potentially driven by specific hydrophobic interactions. For these, the electrostatic repulsion between surfactant headgroups attached to the protein results in significant unfolding of the protein due to a strong change of the electrostatic landscape from that of the native protein, as previously shown by all-atom MD simulations.^43^ At higher surfactant concentration, the formation of larger SDS assemblies without a specific structure becomes more entropically unfavourable, as these result in the exposure of hydrophobic domains to the aqueous environment. Therefore, the sequential formation of micelle-like structures occurs, and a decorated micelle structure is formed. Although the formation of pearl-necklace structures has been previously reported for ionic surfactants and proteins,^3,43^ no evidence of these structures, within the concentration range investigated here, has been found. It is hypothesised that the driving mechanism for the pearl-necklace structure is similar to that of the decorated micelle model, where the hydrophobic residues of the micelle are preferably adsorbed at the micelle core and the hydrophilic groups remain in contact with the solvent. However, the structural resilience that comes from the two disulphide bonds hinders the unfolding of the protein in the presence of surfactant and this retains a relative compactness in the unfolded state.^24^ On the contrary, proteins that present less conformational restraint and more flexible structures may favour the formation of several surfactant clusters attached to the protein backbone. Thus, the final complex structure is intrinsically linked to the structure, dynamics and conformation of the protein.

## Conclusions

Protein solutions in the presence of SDS show a rich and complex variety of conformations, structures and dynamics. The system composed of hGH and SDS is shown to be no exception, and several interaction mechanisms were observed from sub-micellar surfactant concentrations to well above the CMC. ITC experiments revealed that this multi-step character is finely tuned by the SDS/hGH ratio, as well as the stoichiometry of binding between the protein and surfactant. Fluorescence spectroscopy and circular dichroism shed some light on the changes occurring at the local level (protein core) and structural level (secondary and tertiary structure), where a two-stage denaturation begins with the loss of tertiary structure and, upon completion of this initial stage, the secondary structure of the protein is affected by the presence of surfactant clusters. Also, the parametrisation of the CD results provided information on the denaturation trajectories, confirming the formation of a molten globule intermediate.

The characterisation using contrast variation SANS and NMR spectroscopy provided mechanistic information on the interaction between protein and surfactants. SANS allowed structural information to be accessed for specific parts of the complex, as well as to obtain information of the conformational landscape of the protein upon complexation with the SDS. Interestingly, hGH conformation is initially affected by the surfactant interaction, resulting in a partial unfolding. At higher SDS concentrations, the formation of a decorated micelle complex was unambiguously identified. 1D and 2D NMR spectroscopy helped identify the interaction sites in the protein, where the attraction between the surfactant and hydrophobic amino acids was shown to be one of the main driving forces in the complex formation.

Furthermore, the combination of different methodologies reveals specific information on the protein, complex and surfactant behaviour. Importantly, by linking the results from SANS and NMR spectroscopy with the in-house techniques it was possible to shed some light on the characteristic fingerprints of the transitions occurring in the system, where the results from the advanced methods could be connected to those observed using in-house techniques. The development of this kind of knowledge is a huge benefit in the field, as it facilitates the access to detailed information on the interactions between the amphiphiles and the protein using routine methods.

## Materials and methods

hGH, SDS and deuterated SDS (d-SDS, 96% D) stock solutions were prepared in 10 mM phosphate buffer, pH 7, 0.01 wt% NaN_3_, and mixed at different ratios to reach the desired protein and surfactant concentrations. The pH of samples in H_2_O/D_2_O mixtures and D_2_O was corrected for the real *p*(*H*,*D*) values using Rubinson's protocol.^49^ Protein concentration was determined for every sample using the 280 nm absorption peak, and the protein extinction coefficient and molecular weight, 17 670 M^−1^ cm^−1^, 22 124 Da, respectively.^50^

Intrinsic and extrinsic fluorescence spectroscopy was performed using an Agilent Cary Eclipse Fluorescence Spectrophotometer at 22 °C. Circular dichroism measurements were taken using a Chirascan V100, Applied Photophysics at 25 °C. The results were converted to mean residue ellipticity, *θ*_MR_, using the mean residue molecular weight, cell path length and protein concentration in g L^−1^.^51^ Isothermal titration calorimetry measurements were performed on a VP-ITC Microcalorimeter, MicroCal LLC, at 22 °C.

^1^H and ^1^H–^13^C HSQC NMR spectra were recorded on a 700 MHz Bruker Avance III HD at the Swedish NMR Centre (Sweden) at 25 °C. NMR spectra were processed using the NMRPipe suite and visualised using the CCPNMR suite.^52,53^

SANS experiments were performed on SANS2d at ISIS Pulsed Neutron Source (UK), and on D11 at Institut Laue-Langevin (France).^54,55^ The temperature was kept constant at 25 °C for the duration of the experiment. Data reduction was performed using the standard protocols of each beamline.^56,57^ Instrument resolution was accounted for by smearing the theoretical models using a Gaussian function.^58^ Further details on small-angle scattering theory, general experimental details, and data reduction and analysis can be found elsewhere.^59–61^

Samples for SANS measurements were prepared in different contrast conditions to resolve the structure of the complex.^16,36,60^ An isotopic mixture that satisfies the zero-average contrast condition to probe short-range interactions, such as complex formation, was also included in the investigation.^39,40^

Data were analysed using the indirect Fourier transform (IFT) and model-based fitting.^41,62^ The IFT analysis was performed using GNOM, implemented in the ATSAS package.^32,33^ The resulting pair-distance distribution functions (*p*(*r*)) obtained through the IFT approach were used to elaborate eight *ab initio* low-resolution models using DAMMIN per curve, and these were averaged using SUPCOMB.^33,34^ All models derived from those fits were generated, superposed and rendered using the molecular modelling system UCSF Chimera.^63^ Model-based fitting was performed using SasView 4.2.^64^

Further technical details about sample preparation, experimental methods and data analysis are included in the ESI.†

## Author contribution

A. S.-F., S. U., H. S. and M. W. were involved in the experimental design of this project. C. D. designed and performed the protein NMR spectroscopy experiments with input from A. S.-F. and M.W. Scattering experiments were designed and performed by A. S.-F. and J. E. H., with contributions from A. E. L., S. E. R. and S. P. SANS data analysis was performed by A. S.-F., with contributions from J. E. H. J. P. T. prepared the deuterated surfactants used in this investigation and these were characterised by A. E. L. A. S.-F. wrote the manuscript with contributions from all authors.

## Conflicts of interest

There are no conflicts to declare.

## Supplementary Material

NA-002-D0NA00194E-s001
