# Supplementary material for: An integrative toolbox to unlock the structure and dynamics of protein–surfactant complexes
Source: Nanoscale Adv. 2020 Jul 13;2(9):4011–23. doi: 10.1039/d0na00194e (PMC9417085; doi:10.1039/d0na00194e)
Supplement: NA-002-D0NA00194E-s001 [file NA-002-D0NA00194E-s001.pdf]

Electronic Supplementary Material (ESI) for Nanoscale Advances.
This journal is © The Royal Society of Chemistry 2020

Supplementary Information for:

An integrative toolbox to unlock the structure and dynamics of surfactant-stabilised protein formulations

Adrian Sanchez-Fernandez,[1] Carl Diehl,[2] Judith E. Houston,[3] Anna E. Leung,[3] James P. Tellam,[4] Sarah E. Rogers,[4] Sylvain Prevost,[5] Stefan Ulvenlund,[1,6] Helen Sjögren,[7] Marie Wahlgren.[1,6]

[1]Food Technology, Engineering and Nutrition, Lund University, Box 124, 221 00 Lund, Sweden.

[2]SARomics Biostructures AB, Medicon Village, Scheelevägen 2, 223 81 Lund, Sweden.

[3]European Spallation Source, Box 176, 221 00 Lund, Sweden.

[4]ISIS Neutron and Muon Source, Science and Technology Facilities Council, Rutherford Appleton Laboratory, Didcot, OX11 0QX, UK.

[5]Institut Laue-Langevin, 71 avenue des Martyrs, 38000, Grenoble, France.

[6]EnzaBiotech AB, Scheelevägen 22, 223 63 Lund, Sweden.

[7]Ferring Pharmaceuticals A/S, Kay Fiskers Pl. 11, 2300 København, Denmark.

Corresponding author e-mail address: adrian.sanchez-fernandez@food.lth.se

Materials and methods

Materials

All stock solutions and samples used were prepared in phosphate buffer. For buffer preparation, sodium dihydrogen phosphate dihydrate (Sigma Aldrich, >99%) was titrated with a sodium hydroxide solution (Sigma Aldrich, > 99.99%) to a final phosphate concentration of 10 mM and pH of 7.0, in Milli-Q water (Millipore). Sodium azide (Sigma-Aldrich, 99%) was added to a concentration of 0.01 wt% to avoid bacterial growth. Samples containing deuterated solvent were prepared using deuterium oxide (Sigma Aldrich, 99.9% D) and Milli-Q water following the same procedures as described above. For buffer preparation in D_2O and H_2O/D_2O mixtures, the pH values as read by the meter were corrected for the real p(H,D) following the protocol reported by Rubinson.[1]

Freeze-dried and purified recombinant human growth hormone (hGH) was supplied by Ferring Pharmaceuticals and reconstituted in phosphate buffer. The required concentration of protein differed between the experimental techniques. The protein concentration in each sample was determined using absorption spectroscopy (NanoDrop One/One, Thermo Fisher Scientific; or ND-1000 Spectrophotometer, Saveen Werner AB) by measuring the absorption at 280 nm and subsequently calculating the concentration using the protein extinction coefficient and molecular weight (17670 $M^{-1}cm^{-1}$, 22124 Da).[2]

Protiated sodium dodecylsulfate (SDS, Sigma Aldrich, >99 %) was used as received for the preparation of stock solutions in phosphate buffer. The deuterated version of the surfactant (d-SDS) was prepared by the ISIS deuteration facility with a specific level of tail deuteration of 96%, as this matches the scattering length density of D_2O.

To minimise ageing effects, all of the samples involved in this investigation were used within 72 hours of sample preparation, and no evidence of protein aggregation/precipitation was observed during this time. Samples were equilibrated for at least 30 minutes at room temperature prior to measurement. As an exception, samples for SANS experiments were prepared without sodium azide, as these were measured a short time after sample preparation.

Fluorescence spectroscopy

Steady-state fluorescence measurements were performed using an Agilent Cary Eclipse Fluorescence Spectrophotometer with a 4-position sample stage for cuvettes. The temperature in the room was relatively stable at around 22 ˚C during the measurements. Samples were measured in PMMA cuvettes and the contributions to the signal from buffer and cuvette were subtracted from a control measurement of a buffer sample. The reported values resulted from the average of three different measurements, and the errors were calculated as the standard deviation of those.

Two different protocols were employed to characterise the system: intrinsic tryptophan fluorescence; and pyrene fluorescence assay. A 295 nm excitation wavelength was used to measure intrinsic fluorescence of the tryptophan residue of 14.9±0.6 µM hGH in the presence of SDS. The emission spectra were recorded between 310 and 500 nm using 5 nm excitation and emission slits widths, and an integration time of 0.1 s. The results are presented as the spectral centre of mass (CM), which is defined as:

$$CM = \frac{v_i I_i}{I_i}$$

where I_i is the fluorescence intensity at wavenumber v_i.

The pyrene fluorescence assay was used to determine the CMC of the surfactant solution and critical aggregation concentration (CAC) of the system containing protein and surfactant.[3, 4] Different concentrations of surfactant were measured in the absence and presence of 10.3±0.2 μM of protein. For sample preparation, 10 μL of 100 μM pyrene solution in ethanol was added to an empty vial and the ethanol was gently evaporated under a dry atmosphere. The samples were subsequently added to the pyrene vial to give a final pyrene concentration of 2 μM. Emission spectra were measured between 350 and 500 nm using an excitation wavelength of 335 nm. The excitation and emission slits were set to 5 nm and 2.5 nm width, respectively, with an integration time of 0.5 s.

The results from the pyrene assay are presented as the ratio between the fluorescence intensity of the 383 nm and 372 nm peaks, I_3/I_1. These values were plotted against concentration and the transition from the low horizontal region to the steeply increasing signal on the sigmoidal curve was used to identify the CAC and CMC of each system.

Circular dichroism

CD measurements were taken using a Chirascan V100, AppliedPhotophysics, with a temperature-controlled stage to maintain the temperature at 25 ˚C. Samples for far-UV CD measurements were measured between 250 nm and 190 nm, using 30 nm/min scan speed, 1 nm bandwidth and 0.5 s integration time. Samples containing 6.3 μM hGH were prepared at different surfactant concentrations and loaded into 1 mm path length, 1 cm width, quartz Hellma cells for measurement. Near-UV CD was measured between 310 nm and 250 nm for 63 μM protein at different surfactant concentrations. Samples were measured in 10 mm path length, 1 cm width, quartz Hellma cells, using 60 nm/min scan speed, 1 nm bandwidth and 1 s integration time.

For all measurements, the contributions from the cell and the buffer, although minimal, were subtracted from each sample spectrum and then three repeats were averaged. Circular dichroism data was measured in mdeg and converted to mean residue ellipticity, θ_{MR}, using the mean residue molecular weight, cell path length and protein concentration in g/L.[5] No smoothing of data was performed.

Isothermal titration calorimetry

Calorimetric measurements were performed on a VP-ITC Microcalorimeter, MicroCal LLC. The reference cell was filled with buffer and experiments were carried out at 22 ˚C, temperature at which the enthalpy of micellisation of pure SDS in water shows a minimal contribution.[6] Different concentrations of protein in buffer (37, 50, 72, 131, and 204 μM) were loaded in the 1.42 mL cell and titrated using a sequence of 60 injections (4 μL each) of 60 mM SDS in buffer. An analogous experiment was carried out using buffered D_2O at 39 μM hGH and 60 mM d-SDS to evaluate the effect of isotopic substitution.

Raw data were reduced using the Origin-ITC software, where baseline correction and peak integration were performed using the standard protocols of the software. The integration of the heat flow was performed per injection and, as such, it contains both the endothermic and exothermic contributions. The contribution from protein dilution was subtracted from the results of control experiments. The contribution from surfactant demicellisation and micellisation without the protein were measured for comparison. The final dataset was corrected to present the enthalpic change per mole of SDS versus concentration of SDS or SDS/hGH ratio.

Protein NMR

NMR spectra were recorded on a 700 MHz Bruker Avance III HD at the Swedish NMR Centre (Göteborg, Sweden). The temperature was calibrated to 25 ˚C using a 100% MeOD sample. For each sample, ^{1}H and ^{1}H-^{13}C HSQC spectra were collected. ^{1}H NMR spectra were collected using the Bruker pulse sequence "zgespe" with 2 s recycle delay and 64256 points. Sodium trimethylsilylpropanesulfonate (DSS) was added to a concentration of 100 µM as internal standard and all ^{1}H NMR spectra were normalized to the DSS peak. ^{1}H-13C HSQC spectra were collected using the Bruker pulse sequence "hsqcetfpgpsi2". 2048 and 256 points were collected in direct and indirect dimension with a recycle delay of 1.0 s. A spectral width of 20 and 30 Hz were used in ^{1}H and ^{13}C dimension. The ^{1}H and ^{13}C carrier frequencies were placed on 4.7 and 20 ppm.

^{1}H NMR spectra were processed and visualized using MestreNova NMR with squared cosine apodization function and baseline correction. ^{1}H-^{13}C HSQC spectra were processed using the nmrPipe software suite with solvent suppression, squared cosine apodization function in both dimensions and baseline correction in ^{1}H dimension.[7] ^{1}H-^{13}C spectra were visualized using the CCPNMR software suite.[8]

For protein NMR measurements, four samples were prepared using SDS concentrations of 0, 1.46, 14.6, and 146 mM. The protein concentration for all these samples was 0.389 mM in 10 mM phosphate buffer. The buffered solvent contained 10% v/v of D_2O for locking purposes, which was supplemented with 100 µM DSS and 0.02% v/v sodium azide.

Small-angle neutron scattering

SANS measurements were performed on SANS2d at ISIS Pulsed Neutron Source (Didcot, UK), and on D11 at Institut Laue-Langevin (Grenoble, France).[9, 10] Time-of-flight SANS measurements were performed on SANS2d by placing the front and rear detector at 1.6 m and 4 m from the sample, respectively, and using an incident wavelength range of 1.75 to 16 Å separated by time-of-flight, resulting in a combined q-range of 0.006 to 1 Å^{-1}. Further measurements were performed on D11, a monochromatic, pinhole collimated SANS instrument. Measurements were performed using a wavelength of 5.5 Å and sample-to-detector distances of 1.65 m and 8 m to cover a momentum transfer range from 0.009 to 0.4 Å^{-1}.

Data reduction was performed using the standard protocols of each beamline, where data were corrected for detector efficiency, background noise, sample transmission, and scattering from the empty cell and solvent.[11, 12] These resulting data were provided as one-dimension macroscopic scattering cross-section (I(q), cm^{-1}) versus momentum transfer (q, Å^{-1}). The q-resolution for each experiment was calculated from the instrument configuration and the dq/q values were subsequently used to smear the theoretical models using a Gaussian function.[13] Further details on small-angle scattering theory, general experimental details, and data reduction and analysis can be found elsewhere.[14-16]

Samples were loaded into 1- or 2-mm path length cylindrical quartz Hellma cells and placed in a temperature-controlled sample changer. The temperature was kept constant at 25 ˚C for the duration of the experiment.

Scattering theory and analysis

The scattered intensity of centrosymmetric, monodisperse particles after solvent scattering upon background subtraction can be generally described as:

$$I(q) = N_p {V_p}^2 (\Delta SLD)^2 P(q) S(q)$$

where N_p and V_p are the number of density of the particles and volume of those, respectively, and ΔSLD is the excess scattering length density between the particle and the matrix. P(q) is the form factor of the scatterer and relates to its shape, and S(q) is the structure factor, which describes the interaction between particles.

The use of SANS to study structures and interactions in the colloidal domain has been intensively reported and different approaches have been developed for the investigation of mesoscopic systems. In particular, contrast-variation SANS uses isotopic substitution to manipulate the scattering of the system, taking advantage of the fact that protium and deuterium interact differently with the neutrons, to resolve the complex structure of multicomponent systems.[15, 17, 18] A specific case of contrast variation occurs when the scattering length densities of two parts of the system are the same and therefore the scattering excess, ΔSLD, is effectively zero for those components and no contribution to the scattering is observed. This is defined as the contrast-match approach and allows the scattering from specific parts of the complex to be discerned by discriminating the contribution from others.

In addition to the routine contrast-match method, the zero-average contrast (ZAC) condition has been employed. The ZAC limit allows the composition fluctuations to be decoupled from those due to concentration fluctuations. This approach has been widely employed to study polymer-polymer interactions in complex systems, allowing information on short-range interactions to be extracted, such as the formation of complexes.[19, 20] The ZAC condition is fulfilled when the solvent scattering length density (SLD) satisfies the following equation:

$$SLD_{solv} = \frac{SLD_{hGH} v_{hGH} + SLD_{SDS} v_{SDS} N_{agg}}{v_{hGH} + v_{SDS} N_{agg}}$$

where SLD_{solv}, SLD_{hGH} and SLD_{SDS} are the scattering length densities of solvent, protein and sodium dodecylsulfate, respectively, and v_{hGH} and v_{SDS} are the molecular volumes of protein and SDS. N_{agg} is the number of associated surfactant molecules in the assembly. Therefore, by knowing the N_{agg}, the level of solvent deuteration that satisfies such a condition can be determined.

For the analysis of the SANS data, a systematic procedure that combines two different approaches, namely indirect Fourier transform (IFT) and model-based fitting, have been used. The IFT method can only be applied to the analysis of scattering curves from dilute systems, i.e. where the contribution from the structure factor to the scattering can be neglected.[21] Therefore, data were corrected for interparticle scattering and the structure factor was deconvoluted from the experimental intensity. To do so, data were fitted using a model-based fitting approach. The mathematical model selected to account for the form factor varied depending on the characteristic features of the scattering curve, which will be discussed in the Results Section.[22] The rescaled mean spherical approximation (RMSA) was selected to model the structure factor contribution, as it describes particles interacting electrostatically at relatively low volume fractions.[23] The effective radius (r_{eff}) used for calculating the structure factor was determined as the radius of a sphere with the same second virial coefficient of the scatterer. For a prolate ellipsoid $r_{eff}=(r_{eq}\ r_{po}^2)^{1/3}$, where r_{eq} and r_{po} are the equatorial and polar radius, respectively, used for the calculation of the form factor. Due to the coexistence of protein, surfactant micelles and surfactant-protein complexes, this approach provides a mean-field evaluation of the interparticle scattering where the interaction profile is approximated as a single-averaged effect. It is hypothesised that this approach is valid on the basis that the interacting particles are structurally similar, and the volume fractions are relatively low. Therefore, the experimental scattering cross-section is divided by the calculated structure factor, resulting in a corrected scattering pattern that is a function of concentration, composition and morphology of the scatterers.

Initially, SANS data were analysed using the GNOM software implemented in the ATSAS package, which uses the IFT method.[24, 25] The IFT method is a real-space analysis approach for small-angle scattering data, used to obtain the pair-distance distribution function, p(r).[21, 26] The p(r) represents a histogram of the dimensions within the scatterer and is defined by the maximum dimension of the particle, D_{max}, and the restraints p(r)=0 for r=0 and D_{max}>0. Structural information of the particle can be determined from the p(r): the particle radius of gyration, R_g, scattering at zero angle, I(0), and qualitative information of particle morphology, which is extracted from the shape of the function. Once the pair-distance distribution functions were calculated, *ab initio* low-resolution models were determined using the DAMMIN program of the ATSAS package for direct observation of the structure of the scatterer.[24, 27] Eight different models were calculated for each scattering curve and averaged using SUPCOMB from the ATSAS package.[24] All models derived from those fits were generated, superposed and rendered using the molecular modelling system UCSF Chimera.[28]

Finally, detailed structural models of the surfactant-protein complexes were elaborated by co-refining all neutron contrasts using mathematical models implemented in SasView 4.2. Models were continuously refined with the information obtained through IFT and low-resolution bead modelling to converge to a final result.

Synthesis and characterization of d-SDS

Ideally, the SLD of d-SDS should be close to that of D_2O. The deuteration level of SDS required for contrast matching D_2O was calculated using the calculated scattering length densities (SLD) of those components. The theoretical SLD were calculated using the sum of the atomic scattering lengths (b_i), which accounts from the interaction of each group of atoms to the scattering, and the volume they occupy (V_m), using the equation SLD= $\Sigma b_i/V_m$. Neutron scattering lengths for each atom and isotope can be found elsewhere. Values are included in Table S1.

Table S1. Scattering lengths, molecular volumes and theoretical SLD of each component in the system.

Component	b_i / fm	V_m / Å^3	SLD / ×10^{-6} Å^{-2}
$C_{12}H_{25}$	-13.72	352[a]	-0.39
$C_{12}D_{25}$	246	352[a]	6.98
SO_4	26.1	60[b]	4.32
D_2O	19.1	29.9	6.37

[a]The volume of surfactant tails were calculated using Tanford equation.[29]
[b]The volume of the sulfate headgroup was reported by Lu *et al.*[30]

As the surfactant headgroup is significantly hydrated, the contribution to the scattering mainly comes from the tail region of the micelle.[31] Using the SLD presented in Table S1, it was calculated that an approximate tail deuteration level of 93% was required to effectively contrast match the surfactant contribution.

For the synthesis of deuterated sodium dodecyl sulfate, dodecanol-d_{25} (~95 %D, 17.3 g, 81.9 mmol) was dissolved in diethyl ether (dried over sodium, 200 mL) and cooled to 0 ˚C. Chlorosulfonic acid (8.71 mL, 131 mmol) was added dropwise; the temperature remained at 0 ˚C throughout the addition. When the addition was complete the solution was stirred for 1 hour, then added dropwise to an aqueous 2.5 M NaOH solution at 0 ˚C. After the addition, the pH was adjusted to 7 using aqueous 1 M NaOH or aqueous 1 M HCl as required. The ether was removed *in vacuo* and the remaining solution was freeze-dried. The residue was suspended in ether and filtered to remove any remaining dodecanol-d_{25}. The crude product was recrystallised from ethanol to obtain the pure surfactant as a white, crystalline powder (10 g, 40%).

The overall deuteration level of d-SDS was determined by nuclear magnetic resonance (NMR) and electrospray ionization mass spectrometry (ESI-MS). Mass spectra were recorded on an Agilent 1260 Infinity II system coupled with an Agilent InfinityLab LC/MSD XT, in negative mode. The column was by-passed, and the sample was pumped into the mass spectrometer with 1:1 acetonitrile/H_2O + 0.1% formic acid. The overall deuterium incorporation across the molecule was calculated by mass spectrometry using the relative distribution of the isotopologues. This calculation accounted for the ^{13}C natural abundance by subtracting this contribution from the peak area of each M+1 isotopologue. NMR spectra were recorded on a Varian Unity INOVA 400 MHz spectrometer with a 5 mm $^{1}H/^{13}C$ auto-switchable gradient-probe, operating at 400 and 100 MHz respectively for proton (^{1}H) and carbon (^{13}C) nuclei. Spectra were recorded at 298 K. Chemical shifts, expressed in parts per million (ppm), were referenced to the residual signal of the solvent, dimethyl sulfoxide-d_6. The deuterium incorporation at each of the chemically equivalent positions (and from this, the overall deuterium incorporation across the molecule) was determined from the ^{1}H NMR spectrum by comparing the integration of an internal standard, dimethyl sulfone, with the residual protium signals of the deuterated sample. The d-SDS characterization showed that the level of deuteration was 96±2.0% from NMR results and 96±2.0% from ESI-MS results.

The presence of any residual excess scattering length density in the system would result in effective scattering coming from the surfactant phase, which is undesired in the contrast match condition. The level of deuteration obtained from the synthesis was close to the theoretically calculated as the contrast match point. To experimentally confirm whether the contrast match condition was fulfilled by the synthesised d-SDS and no effective scattering in the small-angle was observed, 50 mM d-SDS were measured in buffered D_2O. The results are presented in Figure S1 together with the scattering from 50 mM h-SDS in buffered D_2O and pure buffer for comparison.

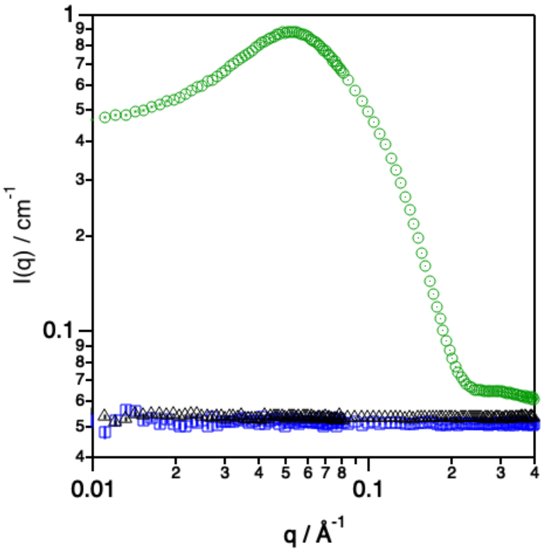

Figure S1 Comparison of scattered intensity of (black triangles) 50 mM d-SDS in buffered D_2O, (green circles) 50 mM h-SDS in buffered D_2O and (blue squares) buffered D_2O. The inset shows the expanded region where the scattering from the contrast-matched surfactant can be better compare to that of D_2O. Data collected on D11.

As it is observed in the experimental data, the signal from the deuterated surfactant is significantly reduced compared to that of h-SDS. The signal from d-SDS completely vanishes in D_2O becoming flat in the entire measured q-range. This signal is practically identical to that

of buffered D_2O, with a slightly higher incoherent background (~3% more), indicating that the contrast match condition is fulfilled.

Isothermal titration calorimetry results

The titration of protein solutions with surfactants leads to characteristic changes in the enthalpograms as measured by isothermal titration calorimetry (ITC). Due to the complexity of the changes occurring upon surfactant addition, as introduced in the main text, the thermodynamic response is often the result of several underlying changes and deconvoluting those becomes challenging. The raw data of the titration of 50 μM human growth hormone (hGH) with 60 mM SDS in 10 mM phosphate buffer is presented in Figure S2.

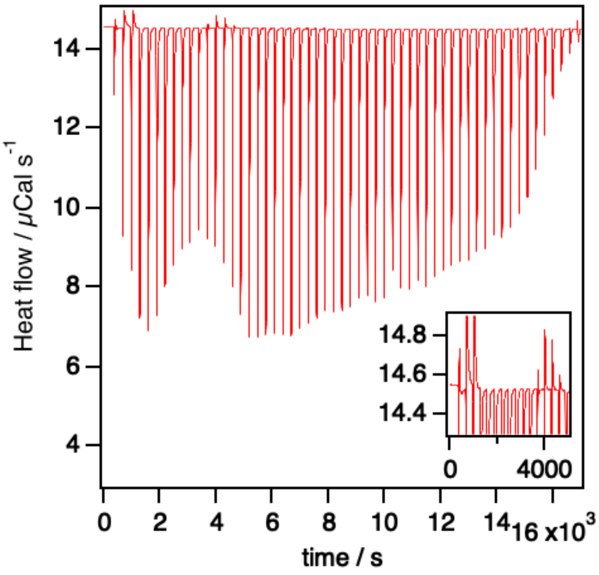

Figure S2 Raw data from the titration of 50 μM hGH with 60 mM SDS in 10 mM phosphate buffer at 22 ˚C. The calorimetric data has been recorded for 60 injections of 4 μL SDS solution. An equilibration time of 300 s was allowed between injections. The inset shows the expanded region from early injections.

In the raw data from the calorimetry experiment it can be seen that some of the individual injections result in multiple peaks. For example, the initial injections (<1500 s) are composed of initially a response above the baseline (~14.5 μCal s^{-1}), immediately followed by a strong contribution below the baseline. This type of behaviour may be indicative of changes occurring in parallel upon injection of surfactant, e.g. surfactant adsorption coupled to protein unfolding.[6]

As presented in the main text, ITC experiments may be used to determine the stoichiometry of binding, where specific features in the enthalpogram relate to certain transitions within the system, e.g. binding saturation and protein conformational changes. Using a mass conservation approach, the total SDS concentration will be distributed between SDS bound to the protein and free SDS in solution. Considering that the driving forces of the interaction are unchanged at different protein concentrations, the stoichiometry of binding can be obtained by running a series of titrations at different titrand concentrations.[32] The enthalpograms of the titrations at different hGH concentration with a 60 mM SDS titrant solution are presented in Figure S3.

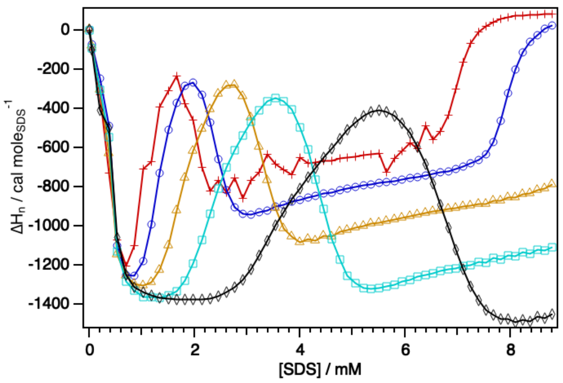

Figure S3 Enthalpograms of the titration of different concentrations of hGH with 60 mM SDS in 10 mM phosphate buffer at 22 ˚C: (red crosses) 37, (blue circles) 50, (yellow triangles) 72, (cyan squares) 131, and (black diamonds) 204 µM hGH.

The ITC protocol used here accessed SDS concentrations between 0 and 8.8 mM and limits the accessible concentration window for the hGH concentrations investigated here. Although this approach presents some advantages, such as a high resolution at low SDS concentrations, it does not reach saturation for all the concentrations investigated here. As such, the results from the binding study were obtained as it follows: the values obtained for transitions i, ii and iii were obtained using 5 data points, iv was calculated from 3 data points, and v using 2 data points. The values were further validated by the SANS analysis of the Surfactant Contrast (see Conformational landscape section from the main text)

Each titration curve can generally be depicted using the same characteristics, where each of the transitions happen at different surfactant concentration. By identifying the surfactant concentration at which each of these occur, the stoichiometry of SDS bound to hGH and the concentration of free SDS can be calculated as presented in the main text.

Intra- and inter-particle scattering in surfactant-protein complexes

The association of SDS with the protein resulted in an increase of the net charge of the surfactant-complexes compared to that of the native protein. As a result of this, interparticle interference appeared on the scattering curve as a smeared peak at q~0.05 Å^{-1} and, thus, it requires to be appropriately treated to extract structural information from the SANS data. As presented in the main text, model-based fitting was the first approach to analyse that data of protein surfactant complexes. Initially, the data from Complex Contrast (surfactant-protein contrast matched) was fitted using a uniform ellipsoid form factor and a rescaled mean spherical approximation (RMSA) structure factor.[23, 33] This approach fits the following parameters to the experimental data: the radius perpendicular (r_{eq}) and along (r_{po}) to the rotational axis of the ellipsoid, the volume fraction of the scatterers (Φ), and the charge of the complex (z). The SLD of the scatterer is fixed to 3.09×10^{-6} Å^{-2}, as presented in the Table 2 of the main text. The ionic strength of the medium was determined from the contribution of the buffer and the amount of dissociated SDS. The relative static permittivity of the solvent was set to 78.3.[34] Data and best fits are shown in Figure S5. The results from those fits are presented in Table S2.

Using the results obtained through this approach, the S(q) was deconvoluted from the experimental scattered intensity for subsequent analysis. In brief, the experimental data from Complex Contrast were divided by the calculated S(q) for each sample, resulting in a scattering curve that relates to the intraparticle scattering and, thus, the P(q) of the particle. A modified approach was used to deconvolute the S(q) from Protein Contrast. Although the scattering from Protein Contrast solely contains information on the protein conformation within the complex,

and therefore relates to different P(q) than those in Complex Contrast at equivalent SDS/hGH ratios, the interparticle scattering arises from the electrostatic interactions between complexes and thus the entire complex must be considered in the calculation of the interparticle scattering. Instead of using the P(q) from Protein Contrast to calculate the effective radius (r_{eff}) of interaction, the same r_{eff} as obtained for Complex Contrast at equivalent SDS/hGH was used. This approach resulted in good fits for Protein Contrast and the contribution from the S(q) was subsequently deconvoluted as presented in the Materials and Methods.

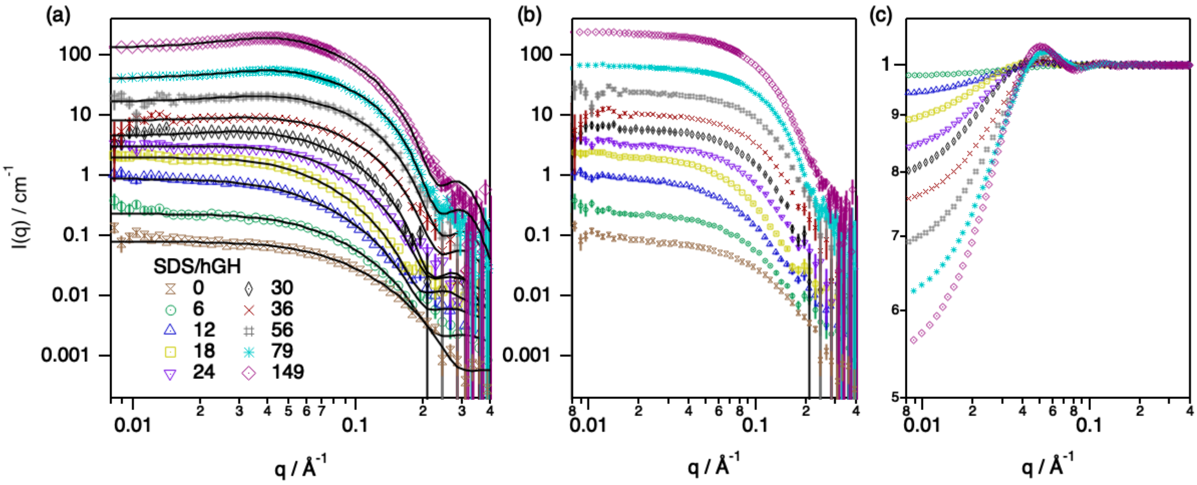

Figure S5 (a) SANS data and best fits from the characterization of Complex Contrast at different SDS/hGH ratios using model-based analysis. Deconvoluted form factor (b) and structure factor (c) resulting from the model-based analysis. These samples were measured using a hGH concentration of 0.135 µM in 10 mM phosphate buffer 100%D and the SDS/hGH ratio is shown in the legend of the graph. Solid lines show best fits to the data. Data and fits in (a) and (b) were offset for clarity. Data were collected on SANS2d and D11.

Table S2 Results from the SANS characterization of Complex Contrast.

SDS/hGH	r_{eq} / Å	r_{po} / Å	Φ / ×10^{-2}	z / -e	I / mM
0	13.7±0.7	40.0±0.7	0.16±0.03	5±2	10
6	17.2±0.3	46.3±1.1	0.13±0.03	8±3	10.8
12	19.4±0.2	56.3±1.0	0.16±0.03	10±4	11.6
18	21.0±0.2	52.8±0.7	0.17±0.02	18±3	12.4
24	19.2±0.2	44.2±0.6	0.18±0.02	28±3	13.2
30	19.1±0.2	38.5±0.6	0.20±0.02	29±2	14.1
36	17.5±0.2	34.4±0.6	0.29±0.03	30±2	14.1
56	17.4±0.2	30.9±0.7	0.31±0.03	29±2	14.1
79	18.2±0.1	29.8±0.2	0.38±0.02	34±2	14.1
149	18.7±0.1	35.6±0.2	0.56±0.02	31±2	14.1

Scattering from SDS micelles

The characterization of SDS micelles in aqueous solution using SANS have previously been reported, showing the formation of globular aggregates that strongly interact in the colloidal domain through electrostatic forces.[35] In order to provide direct comparison to our results, two samples containing 20 and 50 mM SDS in buffered D_2O were measured using SANS. The data was analysed using the exact same protocol used for the protein and surfactant-protein complexes. Initially, the SANS data of SDS micelles were fitted using the uniform ellipsoid model form factor (P(q)) and rescaled mean spherical approximation structure factor (S(q)).[22, 23] Subsequently, the structure factor contribution to the scattering was deconvoluted from that of the form factor, resulting in a scattering curve that was not affected by interparticle scattering. The resulting curve was fitted using the indirect Fourier transform (IFT) approach,

providing the real-space pair-distance distribution function, p(r), of SDS micelles. These results are presented in Figure S4.

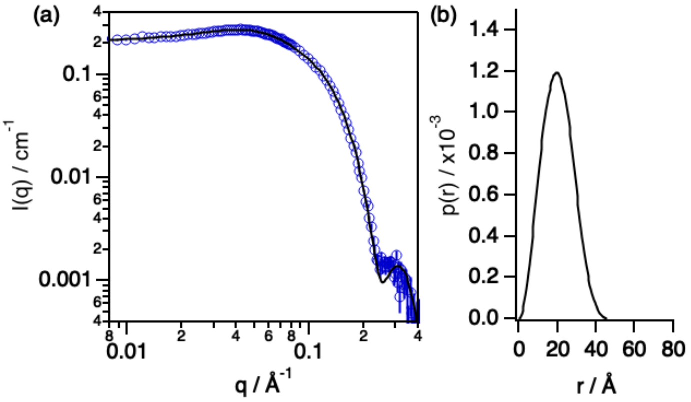

Figure S4 SANS characterization of the scattering from SDS in 10 mM phosphate deuterated buffer. (a) Experimental data and best fits using the model-based analysis. (b) p(r) of SDS micelles as obtained through the IFT approach.

The analysis of the SDS data in buffered D_2O was satisfactorily fitted using the combination of models introduced above, where the micelle can be described as a prolate ellipsoid of revolution with an equatorial radius of 16.8±0.3 Å and a polar radius of 24.4±0.3 Å. From these structural parameters, the aggregation number can be calculated using:

$$N_{agg} = \frac{V}{v_{SDS}}$$

where N_{agg} corresponds to the aggregation number, V is the volume of the micelle and v_{SDS} is the molecular volume of SDS, 407 Å.[30] The resulting N_{agg} for SDS micelles in 10 mM phosphate buffer pH 7 100%D is 71±3. The net charge of the micelle can be calculated using the S(q) model. For the calculation of the Debye length, the ionic strength was set to 14.1 mM, 10 mM from the buffer and 4.1 mM from the surfactant that remains in the monomeric form (CMC), and the relative static permittivity of the solvent at 25 ˚C is 78.3.[34] In these conditions, the calculated net charge of the micelle was 26±3. These values were found to be in similar to the reported structural characteristics of SDS micelles in pure water and the subtle differences may rely on the higher ionic strength of our continuous media.

The structural analysis of the micelle P(q) was also performed using the IFT method as this would allow direct comparison to the structural determinations of the protein and surfactant-protein complexes. From this approach, it was obtained that the maximum dimension of the scatterer (D_{max}) and radius of gyration (R_g) were 48.4±1.0 Å and 15.3±0.2 Å respectively.

Model-based analysis of scattering data

The validity of the structural models of the complexes was further confirmed by using model-based analysis for fitting the data. Two mathematical models were used to simultaneously fit the four SANS contrasts measured. For the low concentration system (SDS/hGH=18) a uniform ellipsoid model was used to fit the data, whereas a core-shell ellipsoid was used to fit the high surfactant concentration samples (SDS/hGH=56).[22] For these models, the equatorial radius (r_{eq}) represents the radius perpendicular to the rotational axis of the spheroid and the polar radius (r_{po}) is the radius along the rotational axis of the ellipsoid. In the case of the core-shell model, these correspond to the structural characteristics of the core, and the thickness of the shell is represented by t_{shell}. The charge of the complex (z) has been determined using the RMSA for fitting the structure factor. From the structural parameters, the surfactant

aggregation number was calculated using the equation presented above. SANS data and fits are presented in Figure 7 of the main text. The results from those fits are included in Table S3.

Table S3 Structural parameters obtained from the co-refinement of the four neutron contrasts using model-based analysis at different SDS/hGH ratios.

SDS/hGH	r_{eq} / Å	r_{po} / Å	t_{shell} / Å	Φ / ×10^{-2}	N_{Agg}	z / -e
18	21.0±0.2	52.8±0.7	-	0.17±0.02	8±6	15±6
56	9.6±0.6	27.6±0.4	8.0±1.8	0.57±0.02	26±5	29±4

Evolution of the surfactant phase in the presence and absence of protein

As presented in the main text, the use of contrast variation allows to determine the structural features of specific parts of the system. The scattering from Surfactant Contrast, where the SLD of the solvent is contrast matched to that of the protein, contains information on the surfactant phase and the structural features of this. The SANS data of this contrasts at different SDS/hGH ratios is presented in Figure S6. Data from equivalent SDS concentrations in a protein-free solution (same deuteration level as Surfactant Contrast) is also shown for comparison.

The SANS data from Surfactant Contrast (and analogous concentrations in the absence of protein) reveal some interesting features. At low SDS concentration, ~0.8 mM, the scattering signal is flat, indicating that no surfactant self-assembled structures are formed. Upon increasing the concentration of SDS, the scattering signal increases in the presence of protein, whilst that remains flat in its absence. This confirms that self-assembly of surfactant at pre-micellar concentrations occur in the presence of 0.135 mM hGH, and the signal arising from these premicellar aggregates increases with SDS concentration. The evidence of SDS self-association in the absence of protein is first observed at 6.8 mM SDS. Although limited structural information can be obtained from this data due to the poor statistics, some qualitative information may be extracted as discussed in the main text.

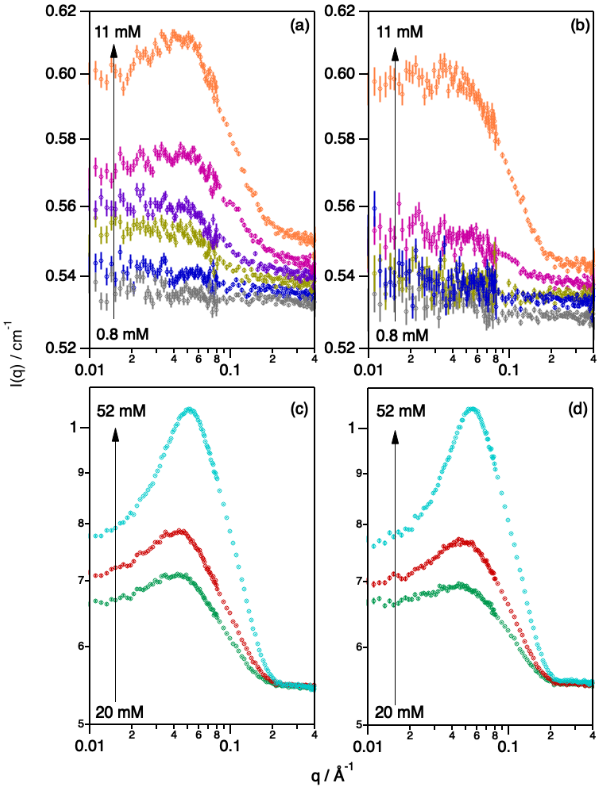

Figure S6 SANS data of the surfactant phase in the system as seen in Contrast 3. Data are shown for (a) low and (c) high SDS concentration in the presence of 0.135 μM hGH, 10 mM

phosphate buffer (41%D). The SANS data from SDS, 10 mM phosphate buffer (41%D), at (b) low and (d) high surfactant concentration in the absence of hGH are presented for comparison. The concentration of SDS varies as indicated in each graph. The solvent contribution to the scattering has not been subtracted from these data. Data were collected on D11.

References

1. K. A. Rubinson, *Analytical Methods*, 2017, **9**, 2744-2750.
2. E. Gasteiger, C. Hoogland, A. Gattiker, S. e. Duvaud, M. R. Wilkins, R. D. Appel and A. Bairoch, in *The Proteomics Protocols Handbook*, ed. J. M. Walker, Humana Press, Totowa, NJ, 2005, DOI: 10.1385/1-59259-890-0:571, pp. 571-607.
3. L. Piñeiro, M. Novo and W. Al-Soufi, *Adv. Colloid Interface Sci.*, 2015, **215**, 1-12.
4. D. Otzen, *Biochim. Biophys. Acta*, 2011, **1814**, 562-591.
5. S. M. Kelly, T. J. Jess and N. C. Price, *Biochim. Biophys. Acta*, 2005, **1751**, 119-139.
6. A. D. Nielsen, L. Arleth and P. Westh, *Langmuir*, 2005, **21**, 4299-4307.
7. F. Delaglio, S. Grzesiek, G. W. Vuister, G. Zhu, J. Pfeifer and A. Bax, *J. Biomol. NMR*, 1995, **6**, 277-293.
8. W. F. Vranken, W. Boucher, T. J. Stevens, R. H. Fogh, A. Pajon, M. Llinas, E. L. Ulrich, J. L. Markley, J. Ionides and E. D. Laue, *Proteins: Structure, Function, and Bioinformatics*, 2005, **59**, 687-696.
9. R. K. Heenan, S. E. Rogers, D. Turner, A. E. Terry, J. Treadgold and S. M. King, *Neutron News*, 2011, **22**, 3.
10. K. Lieutenant, P. Lindner and R. Gahler, *J. Appl. Crystallogr.*, 2007, **40**, 1056-1063.
11. O. Arnold, J. C. Bilheux, J. M. Borreguero, A. Buts, S. I. Campbell, L. Chapon, M. Doucet, N. Draper, R. Ferraz Leal, M. A. Gigg, V. E. Lynch, A. Markvardsen, D. J. Mikkelson, R. L. Mikkelson, R. Miller, K. Palmen, P. Parker, G. Passos, T. G. Perring, P. F. Peterson, S. Ren, M. A. Reuter, A. T. Savici, J. W. Taylor, R. J. Taylor, R. Tolchenov, W. Zhou and J. Zikovsky, *Nuclear Instruments and Methods in Physics Research, Section A: Accelerators, Spectrometers, Detectors and Associated Equipment*, 2014, **764**, 156-166.
12. D. Richard, M. Ferrand and G. J. Kearley, *Journal of Neutron Research*, 1996, **4**, 33-39.
13. J. G. Barker and J. S. Pedersen, *J. Appl. Crystallogr.*, 1995, **28**, 105-114.
14. A. Guinier and G. Fournet, *Small-angle scattering of X-rays*, Wiley, 1955.
15. D. M. Engelman and P. B. Moore, *Annual Review of Biophysics and Bioengineering*, 1975, **4**, 219-241.
16. H. B. Stuhrmann and A. Miller, *J. Appl. Crystallogr.*, 1978, **11**, 325-345.
17. W. Heller, *Acta Crystallographica Section D*, 2010, **66**, 1213-1217.
18. N. R. Zaccai, C. W. Sandlin, J. T. Hoopes, J. E. Curtis, P. J. Fleming, K. G. Fleming and S. Krueger, *Methods Enzymol.*, 2016, **566**, 159-210.
19. M. Benmouna and B. Hammouda, *Prog. Polym. Sci.*, 1997, **22**, 49-92.
20. J. P. Cotton, *Adv. Colloid Interface Sci.*, 1996, **69**, 1-29.
21. O. Glatter, *J. Appl. Crystallogr.*, 1977, **10**, 415-421.

22. J. S. Pedersen, *Adv. Colloid Interface Sci.*, 1997, **70**, 171-210.

23. J.-P. Hansen and J. B. Hayter, *Mol. Phys.*, 1982, **46**, 651-656.

24. M. V. Petoukhov, D. Franke, A. V. Shkumatov, G. Tria, A. G. Kikhney, M. Gajda, C. Gorba, H. D. T. Mertens, P. V. Konarev and D. I. Svergun, *J. Appl. Crystallogr.*, 2012, **45**, 342-350.

25. D. Svergun, *J. Appl. Crystallogr.*, 1992, **25**, 495-503.

26. O. Glatter, *J. Appl. Crystallogr.*, 1980, **13**, 7-11.

27. D. I. Svergun, *Biophys. J.*, 1999, **76**, 2879-2886.

28. E. F. Pettersen, T. D. Goddard, C. C. Huang, G. S. Couch, D. M. Greenblatt, E. C. Meng and T. E. Ferrin, *J. Comput. Chem.*, 2004, **25**, 1605-1612.

29. C. Tanford, *J. Phys. Chem.*, 1972, **76**, 3020-3024.

30. J. R. Lu, A. Marrocco, T. J. Su, R. K. Thomas and J. Penfold, *J. Colloid Interface Sci.*, 1993, **158**, 303-316.

31. J. B. Hayter and J. Penfold, *Journal of the Chemical Society, Faraday Transactions 1: Physical Chemistry in Condensed Phases*, 1981, **77**, 1851.

32. K. K. Andersen, C. L. Oliveira, K. L. Larsen, F. M. Poulsen, T. H. Callisen, P. Westh, J. S. Pedersen and D. Otzen, *J. Mol. Biol.*, 2009, **391**, 207-226.

33. J. Pedersen, *J. Appl. Crystallogr.*, 1994, **27**, 595-608.

34. J. Hamelin, J. B. Mehl and M. R. Moldover, *Int. J. Thermophys.*, 1998, **19**, 1359-1380.

35. J. B. Hayter and J. Penfold, *Colloid. Polym. Sci.*, 1983, **261**, 1022-1030.
